# Bidirectional genetic and phenotypic links between smoking and striatal iron content involving dopaminergic and inflammatory pathways

**DOI:** 10.1111/add.70311

**Published:** 2026-01-31

**Authors:** Olga Trofimova, Ilaria Iuliani, Sven Bergmann

**Affiliations:** ^1^ Department of Computational Biology University of Lausanne Lausanne Switzerland; ^2^ Swiss Institute of Bioinformatics Lausanne Switzerland; ^3^ Department of Integrative Biomedical Sciences University of Cape Town Cape Town South Africa

**Keywords:** caudate, Mendelian randomisation, PascalX, putamen, quantitative susceptibility mapping, T2*, tobacco

## Abstract

**Background and aims:**

Tobacco smoking is a major risk factor for cardiovascular and lung diseases. A better understanding of its neurobiological underpinnings will benefit the prevention of smoking‐related illnesses and mortality. Previous studies link smoking to increased iron concentration in the striatum, a central component of the brain's reward system, and to reduced cognitive performance. This study aimed to investigate whether smoking and striatal iron share common biological pathways and to assess potential causal relationships between the two.

**Methods:**

Using data from the UK Biobank, we investigated phenotypic and genetic correlations, and causal relationships between smoking initiation and magnetic resonance imaging (MRI)‐derived markers of iron content (T2* and quantitative susceptibility mapping) in the bilateral putamen, caudate and accumbens nuclei.

**Results:**

We found positive correlations between smoking and striatal iron (β ∈ [0.03, 0.40], *P* < 0.001), particularly when comparing current smokers with never smokers. Striatal iron was positively associated with pack‐years (β ∈ [0.11, 0.13], *P* < 0.001) and inversely related to years since smoking cessation (β ∈ [0.06, 0.10], *P* < 0.001), suggesting iron levels may decrease after quitting. Genetic analysis confirmed phenotypic correlations, with shared genetic associations (*P* < 2.73 × 10^−6^, or 0.01 for candidate genes) in genes related to dopaminergic, glutamatergic and synaptic systems (*DRD2*, *PPP1R1B*, *NCAM1*, *DLX5*, *GGACT*, *NAT16*, *PLEKHM1*). Causality analysis revealed a relationship from smoking to striatal iron via genes involved in synaptogenesis and plasticity (*BAI3*, *SEMA6D*, *TENM2*), with evidence of reverse causality from iron to smoking through inflammatory and immune system‐related genes (*ING5*, *NLRP7*).

**Conclusions:**

There appear to be links between smoking and striatal iron with complex causal mechanisms involving synaptic transmission and inflammatory circuits. Striatal iron content could serve as a biomarker for smoking‐related neurobiological changes and a potential target for interventions aimed at mitigating cognitive decline related to striatal iron accumulation.

## INTRODUCTION

Smoking is a known risk factor for many diseases, including lung cancer, cardiovascular disease and respiratory disorders. The enormous healthcare costs associated with the adverse effects of tobacco smoking make it a major public health concern. While the effects of smoking on the lungs, cardiovascular system and cancer risk have been extensively studied [1, 2, 3], its impact on the brain has received less attention.

Existing literature has primarily focused on white matter ageing [4, 5, 6] and grey matter volumetry [4, 5, 7, 8, 9, 10, 11, 12, 13], reporting reduced brain volumes and accelerated brain ageing in smokers. Other studies have examined the relationships among smoking, cerebrospinal fluid biomarkers and cognitive impairment [14, 15]. For example, one study found that active smokers had higher Alzheimer’s disease‐related biomarkers, oxidative stress and neuroinflammation [14], while another reported elevated cerebrospinal fluid metals, including iron, and lower cognitive scores correlating with daily cigarette consumption [15]. A few studies have reported increased iron concentration in the striatum of smokers [16, 17, 18].

The striatum is central to motivation, reward, habit formation, addiction and compulsive behaviour, with dopamine and glutamate being the predominant neurotransmitters involved [19]. Striatal dopamine release underpins reinforcement learning and is critical to the addictive effects of substances such as nicotine [19]. Cholinergic modulation via nicotinic acetylcholine receptors also shapes striatal reward processing, mainly through inter‐neuron circuits modulating dopamine release [20, 21]. In this context, iron plays an essential role, particularly in dopamine synthesis, as it serves as a cofactor for tyrosine hydroxylase, the rate‐limiting enzyme in dopamine production [22]. Dysregulation of iron homeostasis in dopaminergic regions such as the striatum has been linked to altered dopamine transmission and receptor availability [23].

Furthermore, iron accumulation in the striatum is associated with lower cognitive performance in older age, increased vulnerability to brain injury from stroke [24] and is a common feature of most neurodegenerative diseases [22, 24, 25, 26]. Processes such as ferroptosis, an iron‐dependent type of cell death, accompanied by iron deposition and glutamate toxicity [24, 26, 27, 28], and iron‐induced neuroinflammation are believed to play roles in Alzheimer’s disease and vascular dementia [29]. More generally, iron is considered an indirect marker of oxidative stress [25, 30, 31].

Given the potential negative clinical consequences of brain iron overload, an important question arises: is there a causal relationship between smoking and iron accumulation in the brain? Existing Mendelian randomisation (MR) studies on alcohol and iron [32], smoking and white matter [6], or subcortical volumes and smoking and alcohol [10] tend to find that behaviour causes changes in brain structure, rather than the other way around. Understanding the biological mechanisms at play could provide crucial insights for clinicians treating nicotine addiction.

This study aimed to investigate the relationship between smoking and striatal iron accumulation at both the phenotypic and genetic levels, assessing potential causal directions and underlying biological mechanisms. Given the role of iron in dopamine synthesis and signalling [22, 26, 31, 33, 34], and the neuroimaging‐based evidence for dopamine increase in the striatum following cigarette smoking [35], we hypothesised that dopamine‐related genes would be jointly involved in smoking and striatal iron. We employed both a candidate gene approach and an exhaustive genome‐wide analysis to test for bidirectional causal relationships.

## METHODS

### Participants

We conducted our study on data from the UK Biobank (UKB), a large‐scale biomedical database that comprises extensive health and genetic information from over 500 000 participants aged between 40 and 69 years at the time of recruitment (2006–2010) [36]. Ethical approval was granted by the National Health Service North West Multi‐centre Research Ethics Committee. All participants provided signed informed consent. We included participants with available brain imaging data and excluded those for whom smoking status was missing. The primary research question and analysis plan for the study were not pre‐registered on a publicly available platform, and the results should therefore be considered exploratory.

### Brain imaging

The UKB brain imaging subsample included 41 844 participants aged from 44 to 85 years at the time of image acquisition [16]. Our study made use of imaging‐derived phenotypes (thereafter referred to as ‘brain traits’) from susceptibility‐weighted magnetic resonance imaging (swMRI) generated by an image‐processing pipeline developed and run on behalf of UKB [37]. More specifically, we examined median T2* and median quantitative susceptibility mapping (QSM) in bilateral putamen, caudate and accumbens nuclei, corresponding to UKB data fields (DFs) 24469–24472, 24479, 24480, 25028–25031, 25038 and 25039. These regions were selected because prior studies have identified elevated iron levels in smokers in the putamen and caudate, and because both dorsal (putamen and caudate) and ventral (accumbens) striatal subregions play a central role in addiction processes [16, 17, 18, 19]. T2* and QSM are swMRI techniques used to assess tissue iron content, with T2* providing a measure of signal decay influenced by iron deposits and QSM offering a quantitative map of tissue magnetic susceptibility to precisely quantify iron levels. Higher iron content results in lower T2* and higher QSM values. Both techniques are also influenced in different ways by other tissue properties, such as myelin, calcium and water, making them complementary [38]. For example, while myelin and iron have the same effect on T2*, they have opposite effects on QSM [18].

### Smoking

Smoking data were self‐reported at the imaging visit. Smoking status (DF 20116) was categorised as current, former or never having smoked. Pack‐years of smoking (DF 20161) was defined as the number of cigarettes smoked per day divided by 20 and multiplied by the number of years of smoking. The number of years of smoking was derived by subtracting the age of starting smoking from either the current age or the age at which smoking was stopped. Years since smoking cessation was calculated as the participant’s age (DF 21003) minus the age at which they stopped smoking (DF 2897).

### Covariates

We selected covariates that are known or are likely to be associated with smoking and brain iron, and could therefore act as potential confounders. General covariates included age (DF 21003), age^2^, sex (DF 31), age × sex, age^2^ × sex, Townsend deprivation index (DF 22189), household income before tax (DF 738), educational attainment (highest among the qualifications reported in DF 6138), alcohol intake frequency (DF 1558), diastolic and systolic blood pressures (mean of the two measurements in DFs 4079 and 4080) and body mass index (BMI; DF 21001). Imaging covariates were included to account for variability in acquisition and scanner effects, following previously published recommendations [39]: assessment centre (DF 54), date (DF 53) and date^2^, protocol change affecting swMRI (DF 24418), head size (DF 25000), scanner and table position (DFs 25756–25759), head motion (DFs 24419, 24441, 24447 and 24 453), variations in acquisition protocols (DFs 25921, 25922, 25923, 25924 and 26500) and intensity scalings (DFs 25925–25930). All covariates were collected at the imaging visit except sex and Townsend deprivation index, which were collected at the first visit. We considered ‘Do not know’ and ‘Prefer not to answer’ as missing values. We imputed missing covariate values by replacing them with the group mean for continuous variables or mode for categorical variables, a simple and widely used approach in large‐scale epidemiological data sets that avoids additional model assumptions (Table S1 shows the number of imputed values). To ensure that this choice did not bias the results, we conducted sensitivity analyses without imputation.

### Phenotypic association between smoking and striatal iron

First, we conducted some data pre‐processing steps. We log‐transformed pack‐years of smoking and Townsend deprivation index, owing to skewness in the data. We excluded outlier values in brain traits beyond five absolute median deviations from the group median (Table S2 shows the number of excluded values). To evaluate whether this exclusion affected the results, we conducted sensitivity analyses without removing outliers. We examined brain traits data distributions, compared male and female means with a two‐sample *t*‐test with Cohen’s *d* for effect sizes, male and female variances with an *F*‐test, and computed Pearson’s correlations between age and brain traits. We also plotted brain trait values stratified by smoking status and sex for visualisation purposes but without statistical testing, as this was investigated later using linear regression models, as described in the next paragraph. Next, on the brain traits and smoking variables, we applied a de‐confounding procedure that consisted of regressing out the above‐listed imaging and non‐imaging covariates, to use the residuals as our new variables. We then performed a rank‐based inverse normal transformation and *z*‐scoring on continuously distributed variables (i.e. de‐confounded brain traits, pack‐years and years since smoking cessation). While the de‐confounding procedure aims to reduce confounding and isolate the biological effects of tobacco smoking, it may also attenuate parts of the total effect of smoking or introduce bias if some covariates act as mediators or colliders. We therefore conducted sensitivity analyses with alternative covariate adjustment strategies to assess the robustness of our findings (see Table S3).

Next, we performed ordinary least squares linear regression models to analyse the relationship between the 12 brain traits (T2* and QSM in the left and the right putamen, caudate and accumbens) and the six smoking variables: ever smoked (current and former smokers vs never smokers), currently smoking (current vs former and never), current versus never (excluding former smokers), current versus former (excluding never‐smokers), former versus never (excluding current smokers) and pack‐years. In all models, the brain trait was the response variable and smoking was the predictor. We also investigated the interaction effects of smoking with sex and age. For former smokers, we ran linear regression models with years since smoking cessation, pack‐years and their interaction term as predictors. For all phenotypic association analyses, we applied false discovery rate (FDR) correction for multiple (six smoking × 12 brain phenotypes) testing [40]. We set the alpha threshold at 0.05 on FDR‐corrected *P*‐values. As a *post hoc* analysis of the left–right asymmetry found in the accumbens QSM associations with the six smoking variables, we compared the respective *β* coefficients from the left and right hemispheres using a *z*‐test.

To systematically compare the alternative models from the sensitivity analyses with our baseline results, we assessed robustness using Pearson’s correlation of *β* estimates and the Jaccard index of significance concordance (defined as the number of associations significant in both analyses divided by the total number significant in either analysis).

### Genetic correlation between smoking and striatal iron

We performed the genetic analyses using publicly available genome‐wide association study (GWAS) summary statistics computed for UKB brain traits from approximately 33 000 unrelated individuals with recent UK ancestry [18, 41, 42]. For smoking, we utilised summary statistics from the larger GWAS and Sequencing Consortium of Alcohol and Nicotine use (GSCAN) study, which included the UKB and 29 other cohorts of European‐ancestry individuals, for a total of approximately 630 000 participants, excluding 23andMe, for which GWAS summary statistics publication is not allowed [43]. We chose ‘smoking initiation’ as our variable of interest, which was the equivalent of ‘ever smoked’ from the phenotypic analysis part of our study, as it is defined for both smokers and non‐smokers and therefore provides a consistent measure of genetic liability to smoking across the entire sample.

We computed the global genetic correlation between each of the 12 brain traits and smoking initiation using linkage disequilibrium score regression (LDSR 1.0.1, Python 2.7) [44] across approximately 1.2 million single‐nucleotide polymorphisms (SNPs). As for the phenotypic analysis, we applied FDR correction [40] to the 12 obtained *P*‐values. We then used PascalX 0.0.3 (Python 3.8.19) cross‐GWAS coherence tests [45, 46] to compute the gene‐wise correlation between smoking and each of the brain traits. PascalX assesses coherent effects across the SNPs associated with two traits within a 50‐kb gene window, accounting for linkage disequilibrium (LD). Here, coherence denotes SNP effects within a gene region influencing both traits in the same direction (similar to a positive genetic correlation), whereas anti‐coherence refers to effects in opposite directions (similar to a negative genetic correlation). We first investigated five dopamine‐related candidate genes expressed in the striatum and previously associated with smoking initiation (*DRD1*, *DRD2*, *DRD3*, *DRD4* and *PPP1R1B*) [34, 43, 47, 48, 49, 50], and then an exhaustive list of 18 344 protein‐coding genes. The LD structure required by PascalX to compute cross‐GWAS coherence scores was provided with the UK10K (hg19) reference panel [51]. Gene annotations were obtained from Ensembl, GRCh37 version (Ensembl release 75) [52]. For positive correlations, we tested QSM coherence and T2* anti‐coherence with smoking, while for negative correlations, we tested QSM anti‐coherence and T2* coherence. Given the brain GWAS sample size, we included in the analysis all SNPs with a minor allele frequency of at least 0.01, which ensured that we got a minimum of approximately 33 individuals with at least one minor allele. We mapped SNPs to genes according to the GRCh37 genome assembly [53]. SNP *P*‐values were rank‐normalised prior to the cross‐GWAS coherence scoring, making the test more conservative but less prone to Type‐1 errors. We also corrected for sample overlap using the LDSR intercept, as the brain GWAS was nested in the smoking GWAS in terms of participants [46]. For comparison, we repeated all PascalX analyses using smoking GWAS summary statistics that excluded UKB participants (*n* ≈ 250 000). We corrected for multiple testing with the Bonferroni method, that is, by setting the alpha threshold to 0.05 divided by the number of genes (18 344 for the exhaustive list of genes and five for the candidate genes). When two or more genes located on the same chromosome, arm and position displayed significant results, we report them here as gene clusters. In such cases, it was not possible to know which gene(s) was (were) driving the signal, given that the gene window we used (50 kb) could be too large for smaller genes.

### Causal relationship between smoking and striatal iron

We investigated potential causal relationships between smoking and striatal iron content with two distinct techniques: Mendelian randomisation (MR) and PascalX cross‐GWAS coherence ratio test [45].

MR is a powerful method commonly used to infer causal relationships between an exposure and an outcome using genetic variants as instrumental variables (IVs) [54]. Here we performed two‐sample MR analyses using the TwoSampleMR 0.5.7 package in R 4.2.2 [55]. We tested causal effects in both directions, assessing whether smoking affects brain iron levels and whether brain iron levels affect smoking, using the inverse‐variance weighted (IVW) method [56] (see the schematic overview in Figure S1). We selected independent SNPs significantly associated with the exposure (*P* < 5 × 10^−8^) as genetic instruments, pruning SNPs with *r* ^2^ > 0.001 to a lead SNP according to LD estimates from the UK10K reference panel [51, 57]. When fewer than five IVs were available, we used a less stringent *P*‐value threshold (*P* < 10^−5^), consistent with previous MR studies under similar constraints [10, 58, 59, 60]. This allowed us to avoid excluding phenotypes entirely owing to insufficient instruments, though this approach may introduce weak instrument bias [61]. To account for this, we repeated all MR analyses using both thresholds. To confirm the validity of the selected SNPs across all trait pairs, we assessed instrument strength using *F*‐statistics. Additionally, we evaluated heterogeneity with Cochran’s *Q*‐test and applied complementary methods robust to heterogeneity, such as MR‐Egger, weighted median or weighted mode, to verify the consistency of causal estimates. We examined MR‐Egger intercepts to detect potential directional pleiotropy. We also conducted leave‐one‐out analysis to ensure that the results were not driven by any single SNP. Finally, we repeated the MR analysis excluding SNPs that had been previously associated with potential confounders, namely alcohol consumption [43] and serum iron [62]. We applied FDR correction for multiple testing [40].

The PascalX ratio test [45] also employs the GWAS summary statistics from two traits. Its test statistic is computed for each gene by summing over the products of the respective effect sizes from all SNPs within a 50‐kb window around the transcribed region of the gene, followed by normalisation by the sum of squared effects from the outcome trait. This approach is conceptually related to MR in that it assumes that a causal effect from an exposure (e.g. smoking) to an outcome (e.g. brain iron) would lead to aligned genetic effects across SNPs near causal genes. The normalisation also ensures that the effects of the exposure that contribute to this correlation must be large (resembling the MR requirement that the instrument variables need to be strongly associated with the exposure), while this is not the case for the outcome. However, unlike MR, which typically relies on genome‐wide significant SNPs as IVs, the PascalX ratio test captures broader polygenic effects while accounting for local LD. As with MR, the validity of causal inference depends on the absence of unmeasured confounding, the directionality of the effect and the exclusion of horizontal pleiotropy (for more details on the ratio test, see [45]). Therefore, we performed a sensitivity analysis excluding genes previously associated with weekly alcohol consumption [43] or serum iron [62] to assess potential confounding (see gene list in Table S4). Although we cannot fully rule out residual confounding effects, this exclusion helps ensure that our findings are not solely driven by known pleiotropic associations. Overall, findings from the ratio test should be interpreted cautiously, and ideally in the context of biological plausibility and complementary evidence. The PascalX parameters were the same as for the cross‐GWAS coherence test, including the Bonferroni correction for the number of tested genes.

## RESULTS

### Participant characteristics and striatal iron distribution by sex, age and smoking status

The phenotypic analysis included 41 844 UKB participants, 22 156 (52.9%) of whom were female. The average age was 64.2 years (±7.7 years). Smoking status was distributed as follows: 3.3% were current smokers, 33.9% were former smokers and 62.8% had never smoked (further covariate descriptive statistics can be found in Table S5).

Females had generally lower iron than males, as reflected by lower QSM and higher T2* values (except in the right accumbens), although the effect sizes were small: |Cohen's *d*| ∈ [0.04, 0.24] (see Figure S2). The variance of brain traits did not differ between males and females (*F*‐values in Figure S2). Iron concentration was higher in older participants in the putamen and caudate, |*r*| ∈ [0.11, 0.33], but did not differ much in the accumbens, |*r*| ∈ [0.02, 0.08] (Figure S3). In both males and females, iron was generally higher in former smokers than in never smokers, and in current smokers than in former smokers (Figure S4).

### Phenotypic association between smoking and striatal iron

**FIGURE 1:**
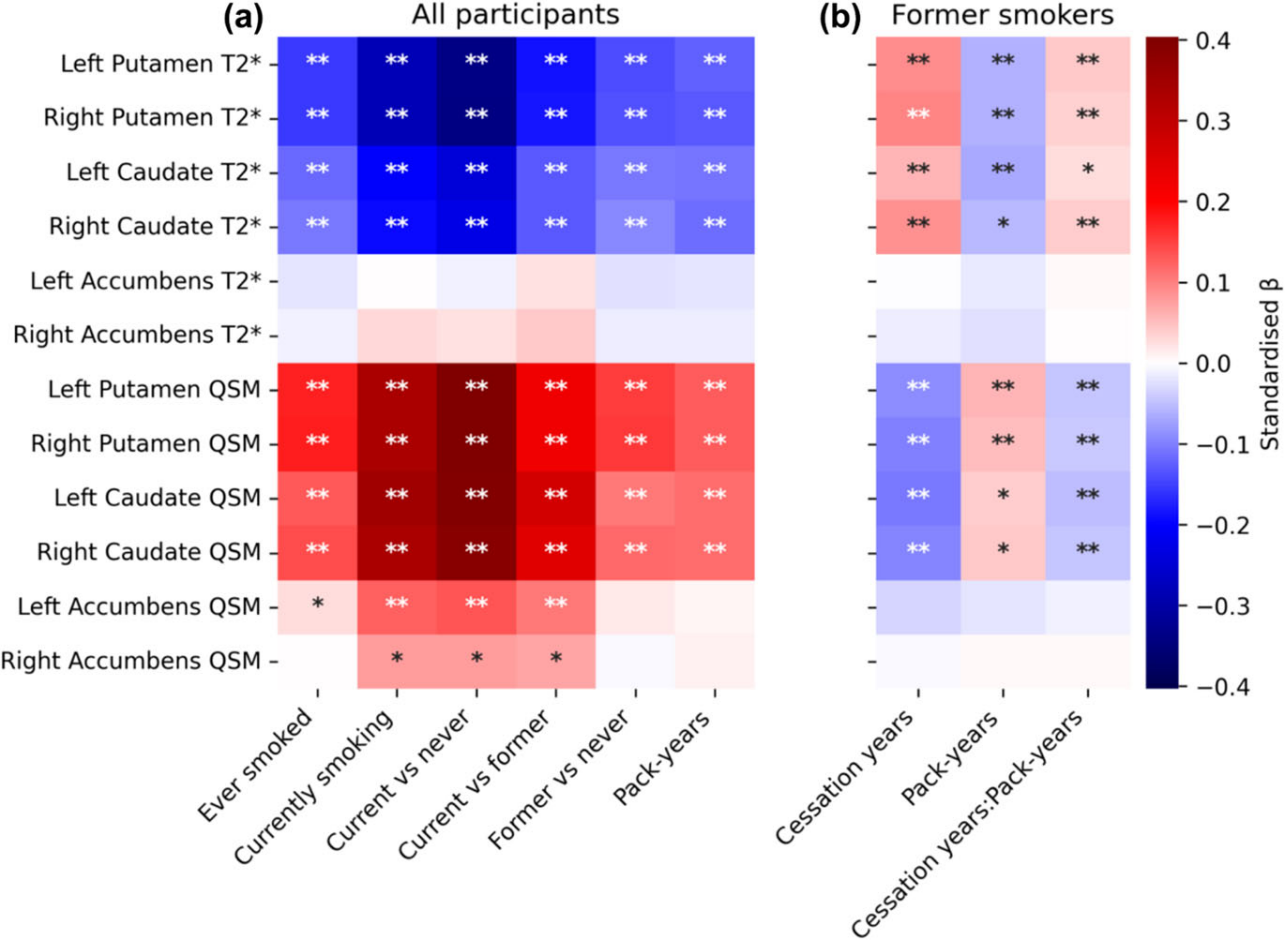
Standardised effect sizes (*β*) of linear regression models linking striatal iron and smoking in (a) the full sample (*n* ≈ 42 000) and (b) former smokers (*n* ≈ 14 000). *β*‐values represent the change in standard deviations of the outcome per standard deviation change in the predictor. *Lower* T2* and *higher* QSM reflect higher iron content. Prior to performing the regression, brain traits and smoking variables were de‐confounded for age, age^2^, sex, age × sex, age^2^ × sex, Townsend deprivation index, income, education, alcohol consumption, blood pressure, BMI and 24 potential imaging confounders (for details, see Methods). *FDR‐corrected *P* < 0.05; **FDR‐corrected *P* < 0.001. BMI = body mass index; cessation years = years since smoking cessation; FDR = false discovery rate; GWAS = genome‐wide association study; QSM = quantitative susceptibility mapping.

Smoking was consistently associated with higher iron content in the bilateral putamen and caudate, as indicated by positive correlations with QSM and negative correlations with T2* (Figure 1a; Table S6). The strongest effects were observed when contrasting current against never smokers: |*β*| ∈ [0.23, 0.40] (corresponding to standard deviation changes in the outcome per standard deviation change in the predictor). In the accumbens, the association with smoking was only present when using QSM as a marker of iron and when contrasting current smokers against other groups, but not former against never smokers, nor when measuring smoking in pack‐years. Interestingly, the association signals in the accumbens were stronger in the left than in the right hemisphere, although the asymmetry was only significant for ‘ever smoked’ (*z* = 1.74, *P* = 0.04; see Table S7). Sex and age interactions with smoking variables were not significant, indicating that the strength of association between smoking and brain traits was similar across ages and sexes (Figures S5 and S6; Table S6). Among former smokers, years since smoking cessation, pack‐years and their interaction were significant in the putamen and caudate but not in the accumbens (Figure 1b; Table S8). For visualisation purposes only, this interaction effect is illustrated in Figure S7 by grouping pack‐years into quartiles: the QSM intercept was higher in individuals with higher pack‐years, the slope associated with years since smoking cessation was generally negative and it was steeper for higher pack‐years values. In other words, the more years passed since smoking cessation, the lower the dorsal striatal iron levels, and the smaller the difference in iron levels between heavy and light smokers. The same was observed with opposite signs in T2*.

Sensitivity analyses using alternative covariate sets, different handling of missing covariates and the inclusion of outliers showed very high consistency with the baseline results (mean *β* correlation = 0.98, mean Jaccard concordance of significant associations = 0.99; see Table S3), indicating that neither covariate selection, imputation nor outlier removal substantially influenced the findings.

### SNP‐level genetic correlation

**FIGURE 2:**
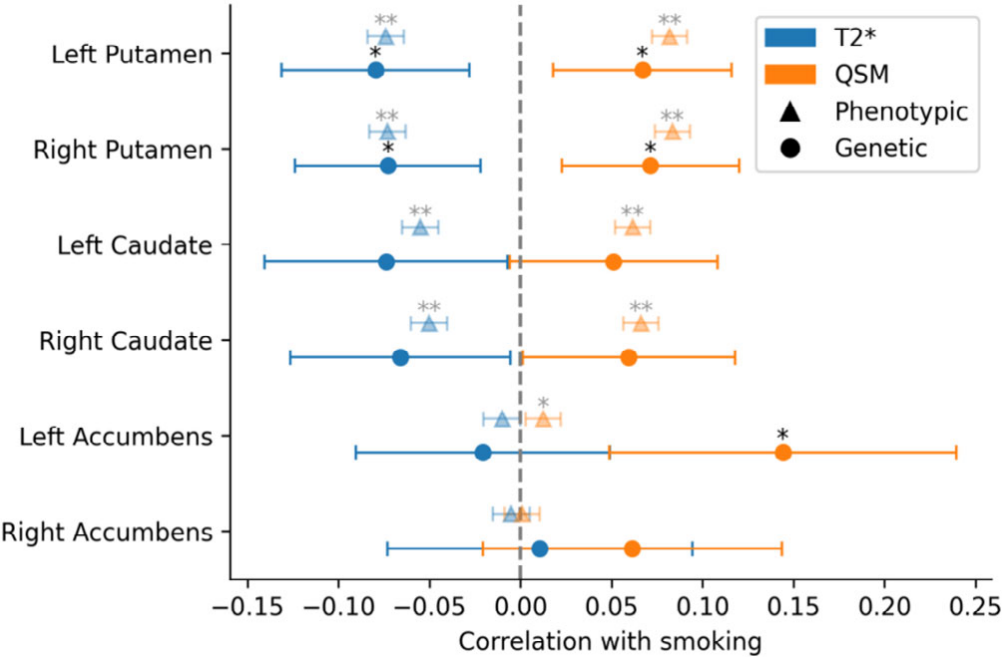
Correlation coefficients between smoking (‘ever smoked’) and striatal iron. Circles represent genetic correlation estimated with LDSR from separate GWAS summary statistics for smoking (*n* ≈ 500 000) and brain traits (*n* ≈ 33 000). *Lower* T2* and *higher* QSM reflect higher iron content. Triangles represent phenotypic correlation between the same variables in the UKB (*n* ≈ 42 000) and are shown for comparison (equivalent to first column in Figure 1 but expressed as correlation coefficients rather than *β*‐values). Error bars represent 95% confidence intervals. *FDR‐corrected *P* < 0.05, **FDR‐corrected *P* < 0.001. FDR = false discovery rate; LDSR = linkage disequilibrium score regression; QSM = quantitative susceptibility mapping.

We observed a positive genetic correlation between smoking and QSM in the bilateral putamen and the left accumbens, *r* ∈ [0.07, 0.14], and a negative correlation between smoking and T2* in the bilateral putamen, *r* ∈ [−0.08, −0.07] (see the circles in Figure 2). These results were consistent with the corresponding phenotypic correlations (Figure 2 diamonds, equivalent to the first column in Figure 1a but expressed as correlation coefficients rather than effect sizes), except for the caudate, where the genetic correlations were no longer significant after correcting for multiple testing. Standard errors were much larger for genetic correlation estimates than for phenotypic correlation estimates (see confidence intervals in Figure 2 and Table S9 for details).

### Gene‐level analysis

**FIGURE 3:**
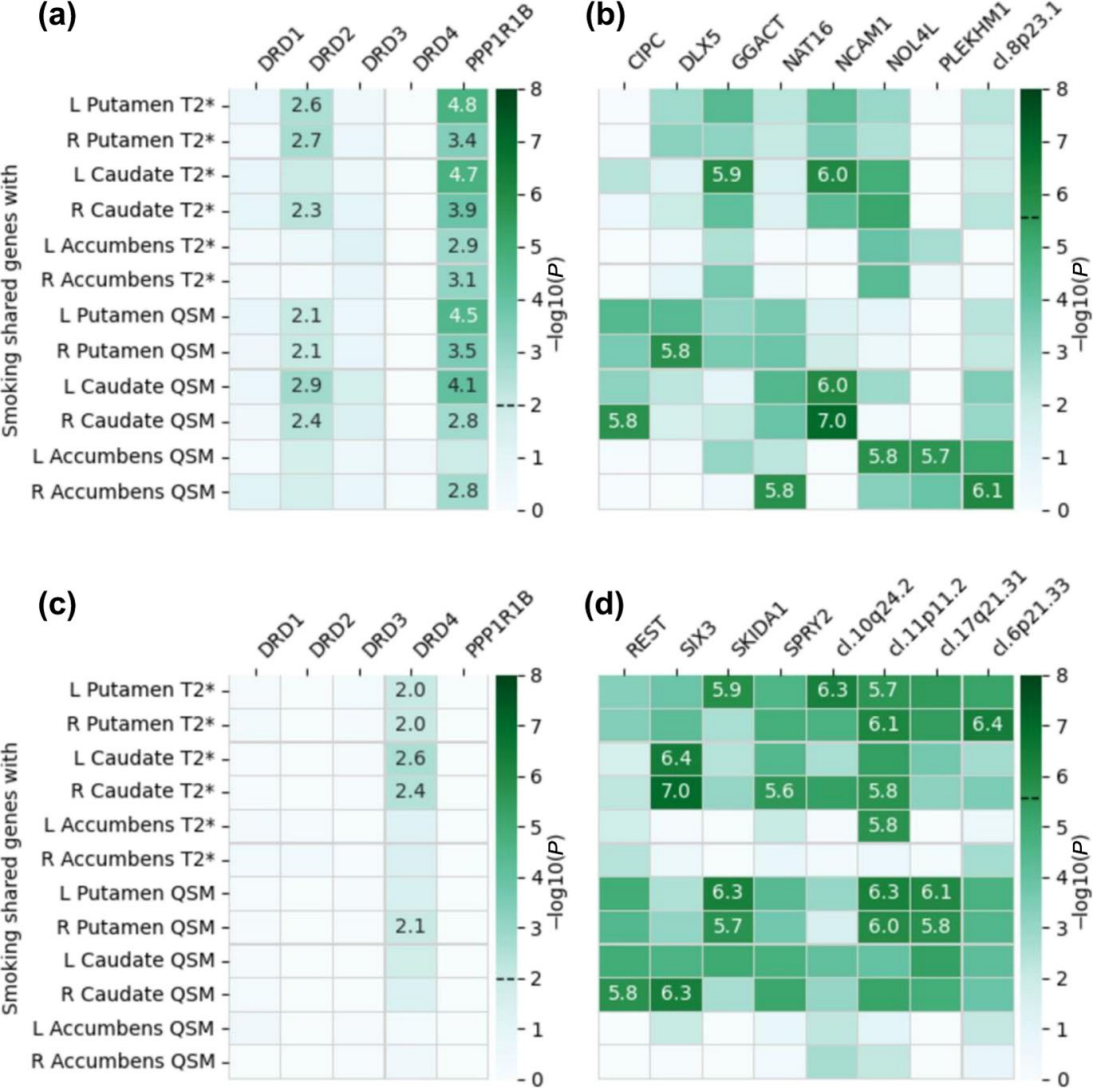
Gene‐level correlation between smoking and striatal iron performed using the PascalX cross‐GWAS coherence test. We tested (a, b) positive and (c, d) negative correlations for (a, c) five dopamine‐related candidate genes and (b, d) an exhaustive set of 18 344 genes. –Log10(*P*) values are annotated for Bonferroni‐significant pairs, that is, paires with *P*‐values below (a, c) 0.01 (0.05/5 candidate genes) and (b, d) 2.73 × 10^−6^ (0.05/18 344 tested genes). Significance thresholds are indicated by dashed lines on the colour bars. L = left; R = right; QSM = quantitative susceptibility mapping. ‘cl.’ indicates gene clusters with their cytogenetic location: cl. 6p21.33—*CLIC1*, *DDAH2*, *LY6G6C*, *LY6G6D*, *LY6G6E*, *LY6G6F*, *MPIG6B*, *MSH5*; cl. 8p23.1—*C8orf74*, *PINX1*, *RP1L1*, *SOX7*; cl. 10q24.2—*AS3MT*, *BORCS7*, *CNNM2*; cl. 11p11.2—*FREY1*, *GYLTL1B*, *PEX16*; cl. 17q21.31—*ARL17B*, *LRRC37A*, *PLEKHM1*.

The PascalX cross‐GWAS analysis revealed positive correlations between smoking and striatal iron for the dopamine‐related genes *DRD2* (in the putamen and caudate) and *PPP1R1B* (all regions, see Figure 3a). In the exhaustive gene set analysis, *NCAM1* was the only gene found for more than one brain region and contrast (bilateral caudate). Other genes included *DLX5* in the putamen, *CIPC* and *GGACT* in the caudate, and *NAT16*, *NOL4L*, *PLEKHM1* and a gene cluster on chromosome region (chr) 8p23.1 in the accumbens (see Figure 3b and Table S10 for detailed results). Those genes are involved in synaptic function and GABAergic and glutamatergic metabolism [63, 64, 65].

A negative correlation between smoking and striatal iron content was found for *DRD4* in the putamen and caudate, a gene cluster on chr 11p11.2 (putamen, caudate and left accumbens), *SKIDA1*, clusters on chr 10q24.2, chr 6p21.33 and chr 17q21.31 in the putamen, and *REST*, *SIX3* and *SPRY2* in the caudate (Figure 3c and d and Table S10). Among other functions, those genes play a role in the immune system, signal transduction and stress response [65, 66, 67].

Sensitivity analysis excluding genes previously associated with potential confounders (alcohol consumption or serum iron) led to the removal of *DRD2*, *PLEKHM1* and *SIX3* owing to their reported associations with alcohol, while the overall pattern of results remained consistent (Figure S8).

### Causal relationship

**FIGURE 4:**
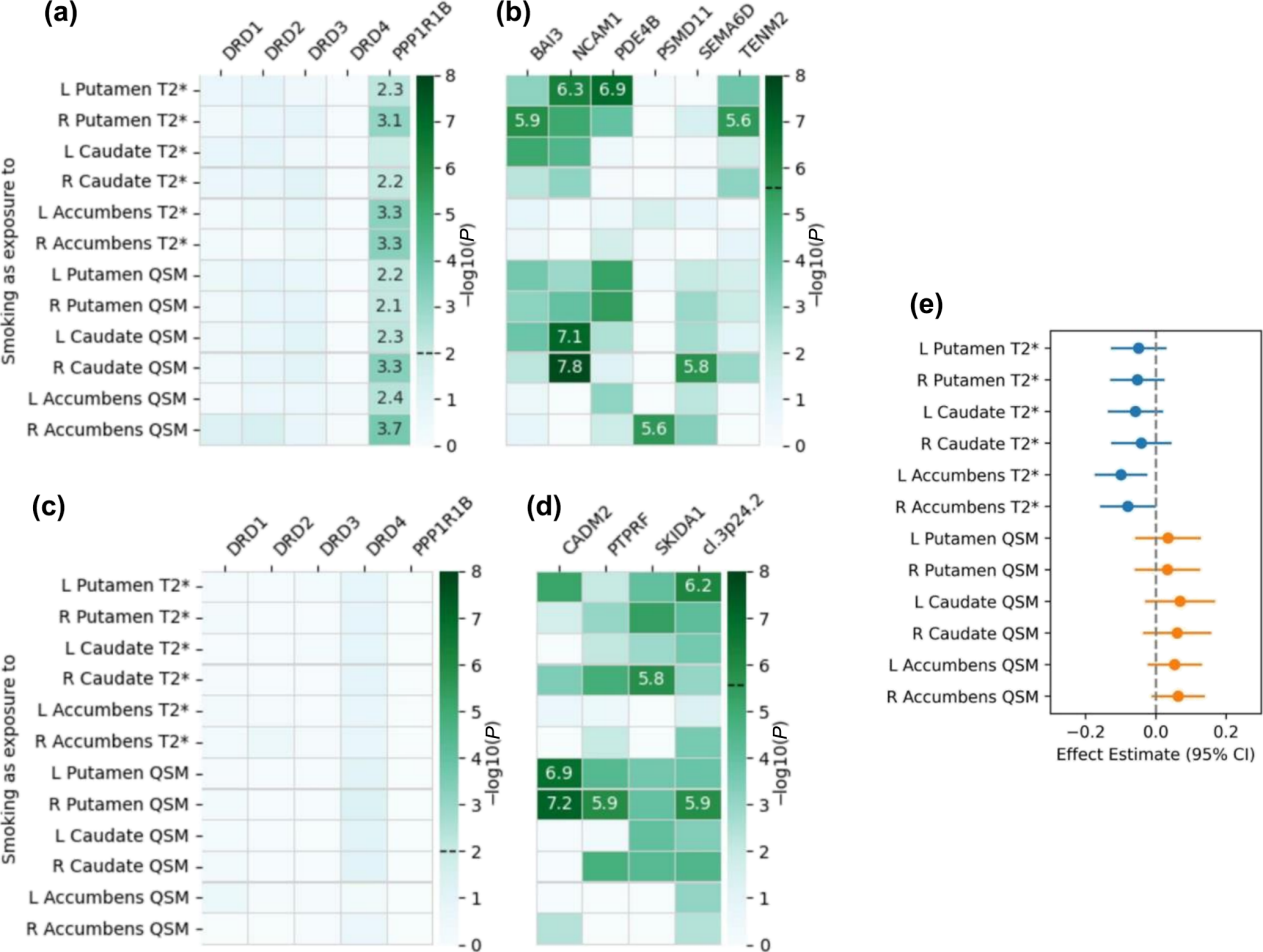
Causality pathway from smoking to striatal iron. For panels (a)–(d) we used the PascalX cross‐GWAS ratio test for (a, b) positive and (c, d) negative causal associations in (a, c) five dopamine‐related candidate genes and (b, d) an exhaustive set of 18 344 genes. –Log10(*P*) values are annotated for Bonferroni‐significant pairs, that is, with *P*‐values below (a, c) 0.01 (0.05/5 candidate genes) and (b, d) 2.73 × 10^−6^ (0.05/18 344 tested genes). Significance thresholds are indicated by dashed lines on the colour bars. (e) Mendelian randomisation IVW estimates between smoking and striatal iron are indicated with their 95% CIs. None of the IVW estimates was significant after FDR correction. FDR = false discovery rate; IVW = inverse‐variance weighted; L = left; QSM = quantitative susceptibility mapping; R = right. ‘cl.’ indicates gene clusters with their cytogenetic location. cl. 3p24.2: *RARB*, *TOP2B*.

**FIGURE 5:**
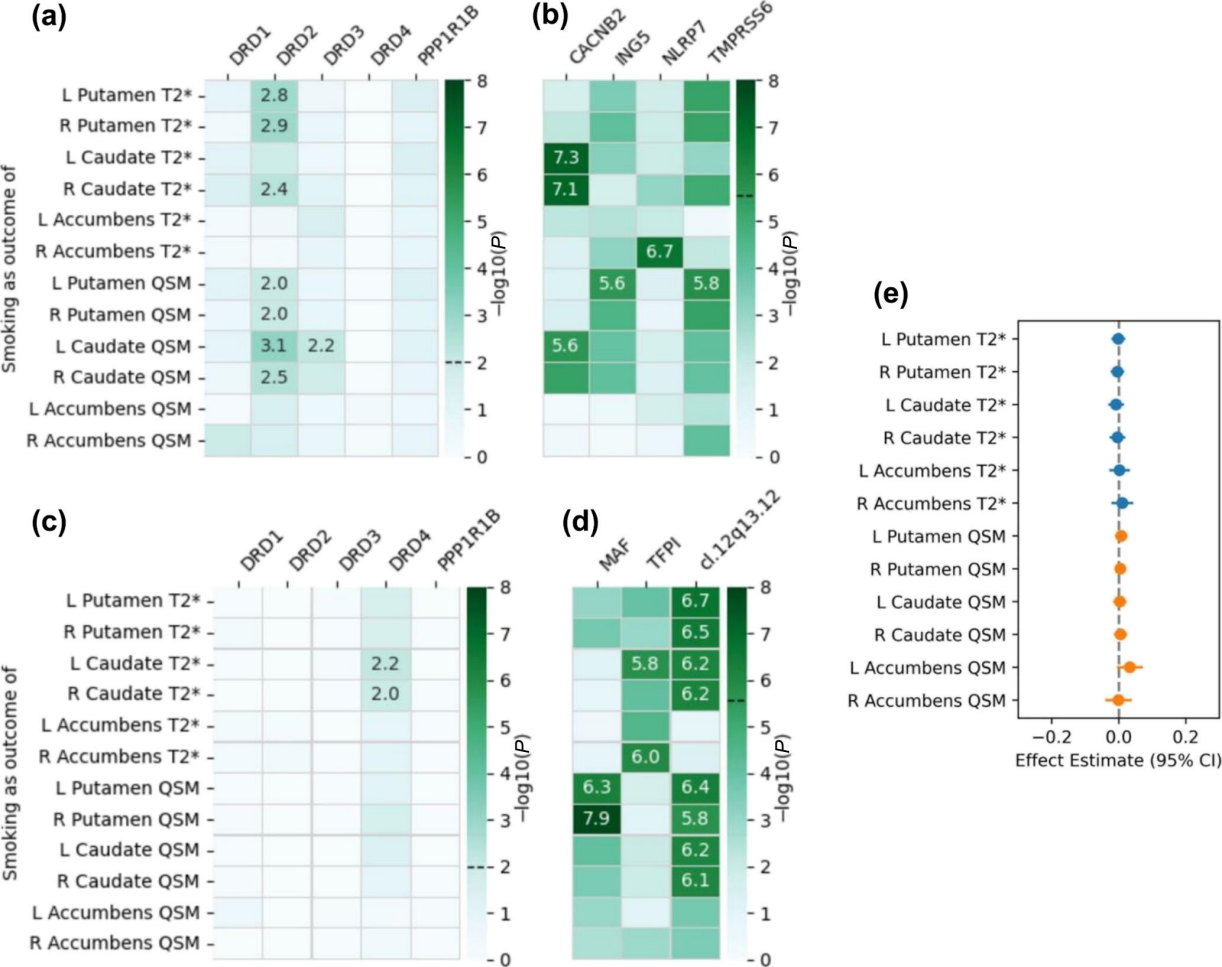
Causality pathway from striatal iron to smoking. For panels (a)–(d) we used the PascalX cross‐GWAS ratio test for (a, b) positive and (c, d) negative causal associations in (a, c) five dopamine‐related candidate genes and (b, d) an exhaustive set of 18 344 genes. –Log10(*P*) values are annotated for Bonferroni‐significant pairs, that is, with *P*‐values below (a, c) 0.01 (0.05/5 candidate genes) and (b, d) 2.73 × 10^−6^ (0.05/18 344 tested genes). Significance thresholds are indicated by dashed lines on the colour bars. (e) Mendelian randomisation IVW estimates between striatal iron and smoking are indicated with their 95% CIs. None of the IVW estimates was significant. FDR = false discovery rate; IVW = inverse‐variance weighted; L = left; QSM = quantitative susceptibility mapping; R = right. ‘cl.’ indicates gene clusters with their cytogenetic location. cl. 12q13.12: *TFCP2, TMT1A*.

Our MR analysis did not yield statistically significant results: |β| ∈ [0, 0.1]; FDR‐corrected *P* ∈ [0.11, 0.96]. In the smoking → iron direction, nominal associations were observed in the bilateral accumbens (*P* < 0.05), but these did not survive correction for multiple testing. No evidence for causality was observed in the iron → smoking direction (Figures 4e and 5e; Table S11). While these findings are not conclusive, they indicate a numerical pattern more consistent with smoking influencing iron than the reverse. All sensitivity analyses were in line with the main results (Tables S11–S14). Because the main MR analysis used smoking summary statistics that included UKB participants, some sample overlap with the brain iron GWAS may have introduced a bias toward inflated effects. However, results obtained with non‐overlapping data (Table S14) showed consistent directionality and similarly non‐significant associations: |*β*| ∈ [0, 0.09], nominal *P* ∈ [0.29, 1]), supporting the robustness of our conclusions.

The PascalX ratio test indicated positive bidirectional causality through genes expressed in brain tissues (detailed results in Tables S15 and S16). Specifically, for the causal pathway from smoking to striatal iron (i.e. smoking increasing iron levels), the implicated genes were *PPP1R1B*, *BAI3*, *NCAM1*, *PDE4B*, *PSMD11*, *SEMA6D* and *TENM2* (Figure 4a and b), which are associated with dopaminergic and glutamatergic activity, synaptic plasticity and neuroinflammation [65, 68, 69, 70]. Conversely, for the pathway from striatal iron levels to smoking (i.e. higher iron increasing smoking risk), the involved genes were *DRD2*, *DRD3*, *CACNB2*, *ING5*, *NLRP7* and *TMPRSS6* (Figure 5a and b), linked to dopamine signalling, iron homeostasis and inflammation [65, 71, 72].

Additionally, the PascalX ratio test identified negative causality (i.e. between smoking and *lower* iron). For the causal pathway from smoking to iron content in the putamen and caudate (i.e. smoking decreasing iron levels), the genes *CADM2*, *PTPRF* and *SKIDA1*, and a gene cluster on chr 3p24.2 (Figure 4c and d) were involved, primarily associated with immune system functions and synapse organisation [65, 73, 74]. For the reverse pathway, from striatal iron to smoking (i.e. higher iron reducing smoking risk), the genes *DRD4*, *MAF* and *TFPI*, and a gene cluster on chr 12q13.12 were implicated (Figure 5c and d), with functions related to dopamine signalling, inflammatory response and the immune system [65, 75, 76].

Sensitivity analyses that excluded genes linked to potential confounders resulted in the removal of *PDE4B*, *TENM2*, *CADM2* and *DRD2* (associated with alcohol consumption) as well as *TMPRSS6* (associated with serum iron), while the overall pattern of findings remained unchanged (Figures S9 and S10). Additional analyses using smoking summary statistics excluding UKB participants yielded broadly consistent results, but with a reduced number of significant genes (Figures S11–S13), consistent with the lower power of this data set.

## DISCUSSION

In this study, we identified a positive association between tobacco smoking and increased iron content in the striatum, particularly in the dorsal striatum (putamen and caudate). Notably, iron accumulation in the dorsal striatum was positively correlated with pack‐years of smoking and negatively correlated with the number of years since smoking cessation, with stronger effects observed in individuals with greater lifetime tobacco exposure. These findings could not be attributed to linear effects of alcohol consumption, blood pressure, BMI, demographic, socio‐economic or imaging variables, as we corrected for these confounders prior to the analysis. Additionally, we observed a global positive genetic correlation between smoking and iron levels in the putamen and left accumbens. Gene‐specific analyses revealed a complex pattern of positive and negative associations between smoking and iron, reflecting diverse genetic pathways. Evidence of bi‐directional causal relationships was found, involving genes related to synaptic function (*BAI3*, *CACNB2*, *CADM2*, *NAT16*, *NCAM1*, *PLEKHM1*, *PTPRF*, *SEMA6D*, *TENM2*), dopaminergic (*DRD2*, *DRD3*, *DRD4*, *NCAM1*, *PPP1R1B*), GABAergic (*DLX5*) and glutamatergic (*GGACT*, *PPP1R1B*) transmission, as well as immune function, inflammation and stress response (*NLRP7*, *MAF*, *REST*, *SIX3*, *SKIDA1*, *SPRY2*, *TFCP2*, *TMT1A*, *TOP2B*, and a gene cluster on chr 6p21.33).

The association of iron levels with smoking in the dorsal striatum appears to be cumulative, as it is proportional to pack‐years, and potentially reversible, given that individuals with a longer history of smoking cessation showed lower iron levels. Although this is speculative given the correlational nature of our results and would require confirmation in a longitudinal design, if true, it suggests that smoking cessation could have a positive impact on brain health, similar to the gradual recovery of lung function observed after smoking cessation [77]. In contrast, iron levels in the ventral striatum (accumbens) were elevated only in current smokers and showed no correlation with pack‐years. This might be linked to the differential dopamine signalling responses to nicotine between the ventral and dorsal striatum, limiting long‐term effects primarily to the dorsal striatum [78]. Research indicates that the ventral striatum initially drives voluntary drug use, while the dorsal striatum increasingly takes over as habitual patterns emerge, facilitating the progression to compulsive behaviour during habit formation [79]. The leftward asymmetry that we observed in the accumbens could be interpreted in light of disrupted accumbens volume asymmetry reported in a recent multi‐cohort study, where nicotine‐dependent individuals had larger left accumbens volumes than non‐dependent controls [80]. The observed link between smoking and left accumbens iron using QSM, but not T2*, could suggest differential sensitivity to tissue compartments such as myelin. However, T2* measurements in the accumbens have been shown to be less reliable than in the caudate or putamen, or than QSM in the same region, with lower cross‐scan reproducibility and higher susceptibility to artifacts arising from macroscopic field gradients [18]. These methodological limitations may obscure true associations and should be considered when interpreting null results in the accumbens T2*.

Iron accumulation in the brain may be linked to ferroptosis, which has been linked to inflammatory pathways [81]. Our identification of genes involved in oxidative stress, inflammation, and immune response aligns with the hypothesis of heightened ferroptosis and inflammation related to smoking. This finding is particularly important given that smoking is a known risk factor for Alzheimer’s disease and other neurodegenerative diseases, where oxidative stress and neuroinflammation have been specifically identified as key mechanisms [14, 26, 28].

Mendelian randomisation (MR), a well‐established tool to investigate causality, did not produce significant results, although there was a trend indicating that smoking influences iron levels rather than vice versa. This trend aligns with previous research linking substance dependence to brain MRI measures [6, 10, 32]. Although some sample overlap in GWAS summary statistics could have introduced bias, the consistency with the non‐overlapping data set (reported in the supporting information) strengthens confidence that the observed null effects are genuine. One likely explanation for why PascalX detected associations that MR did not is that PascalX distinguishes between coherent and anti‐coherent gene signals (e.g. smoking increasing vs decreasing iron), whereas such opposing effects would largely cancel each other in MR. Moreover, smoking behaviour and brain iron are highly polygenic traits influenced by numerous factors, making it difficult to detect causal effects using the limited number of genome‐wide significant SNPs typically employed in MR. In contrast, by aggregating SNP‐wise signals at the gene level, PascalX was able to capture broader polygenic effects. Consistent with the observed MR trend, PascalX identified more genes with smoking as exposure and iron as outcome than in the reverse direction. As we discuss in the following, the vast majority of genes detected with PascalX appear functionally plausible. This suggests that PascalX can serve as a powerful extension of standard MR, particularly in settings where results are inconclusive or only marginally significant.

We hypothesised that the relationship between smoking and striatal iron would involve the dopaminergic system. Two dopamine receptor genes, *DRD2* and *DRD4*, were identified with opposing effects in the dorsal striatum. *DRD2* (along with *DRD3* in the causal analysis) was genetically associated with smoking and higher iron levels in the dorsal striatum, whereas *DRD4* was linked to lower iron. Both genes were implicated in the causal pathway from striatal iron to smoking, suggesting that an imbalance in striatal dopamine receptors may increase the likelihood of initiating smoking. It should be noted that *DRD2* has also been previously associated with alcohol consumption, raising the possibility that our findings partly reflect confounding. The idea that brain iron content could influence behaviour may appear speculative, but it is supported by pre‐clinical evidence. Animal studies have shown that both iron deficiency and iron overload can modulate reward sensitivity and affect susceptibility to drug‐seeking behaviour [82, 83]. For instance, induced iron deficiency in rats reduces the motivation for cocaine [84], while promoting brain iron efflux has been shown to lower brain iron levels, increase dopamine transporter expression, and reduce the rewarding effects of morphine and methamphetamine [85].

*PPP1R1B*, a gene that regulates dopaminergic and glutamatergic signalling and plasticity in the striatum, showed coherent signals for smoking and iron content, with a causal direction from smoking to iron accumulation. A similar pattern was observed for *NCAM1* in the dorsal striatum. *NCAM1* has previously been identified as a binding partner of *DRD2* and a modulator of dopaminergic activity, forming a complex with *DRD2* upon dopamine stimulation, particularly through its *NCAM180* isoform [86]. The *NCAM1* gene is part of a cluster (*NCAM1–TTC12–ANKK1–DRD2*) implicated in various dopamine‐related disorders, including attention deficit hyperactivity disorder and substance dependence [87, 88, 89]. Our findings expand this understanding by suggesting a causal relationship in which smoking behaviour leads to iron accumulation in the striatum via stimulation of the dopaminergic system.

Beyond genes involved in dopamine transmission, we identified several genes linked more broadly to synaptic function, predominantly in the causal pathway from smoking to dorsal striatal iron. This suggests that synaptic plasticity in smokers may contribute to increased iron content in the dorsal striatum. Alternatively, specific synaptic organisation during development could predispose individuals to smoking, with iron accumulation occurring as a downstream effect. Several genes previously associated with substance abuse beyond cigarette smoking (*BAI3*, *CADM2*, *ING5*, *PSMD11*) [90, 91, 92] and, more generally, with impulsive behaviour and risk‐taking (*BAI3*, *CADM2*, *PTPRF*) [93] point to potential mechanisms, such as synaptogenesis and signalling (*BAI3*, *CADM2*) [92, 94], increased acetylation of histones H3 and H4 within the reward circuitry (*ING5*) [95], and protein–protein interactions at synapses (*PTPRF*) [73].

Our study has several limitations. The phenotypic analysis was conducted primarily in samples of White European ancestry, and the genetic analysis was limited exclusively to European ancestry, which restricts the generalisability of our findings. Although a recent multi‐ancestry smoking GWAS has been published [96], there is currently no large‐scale GWAS of brain iron markers in diverse populations. Such data sets will be crucial to replicate our findings across ancestries and to confirm whether the same genetic mechanisms are at play in non‐European populations. Ideally, the genetic analysis would be validated in an independent cohort, but this was not feasible as the UKB is the only study of sufficient size that includes brain swMRI data. Additionally, the UKB cohort is relatively old, and younger adults are not represented. As iron accumulation in the striatum is strongly age related, the effects observed here may be less pronounced in younger populations, limiting the generalisability of our results across the full adult lifespan. Finally, while we controlled for potential confounders in the phenotypic analysis, it was not possible to achieve the same level of control in the analyses relying on GWAS summary statistics from previous studies. As a result, LDSR genetic correlations could still be influenced by other substance use or unmeasured confounders. Although our sensitivity analyses excluding SNPs and genes previously associated with alcohol consumption and serum iron yielded similar conclusions, we cannot fully exclude the possibility of residual confounding effects in the genetic findings.

In summary, our study reveals a complex relationship between smoking and striatal iron levels, with overall positive genetic and phenotypic correlations. Gene‐level analyses suggest a trend where smoking primarily influences iron levels, although both positive and negative associations were observed, indicating intricate bidirectional mechanisms and possible feedback loops. Our findings suggest that smoking may lead to increased striatal iron accumulation through multiple pathways, which may impact brain health and neurodegenerative risk. Additionally, inflammation and dopamine imbalance in the striatum may further reinforce smoking behaviour. Further research, particularly involving more diverse populations and longitudinal data, is essential to fully elucidate these dynamics.

## AUTHOR CONTRIBUTIONS

**Olga Trofimova:** Conceptualization (equal); data curation (lead); formal analysis (lead); methodology (equal); visualization (lead); writing—original draft (lead); writing—review and editing (equal). **Ilaria Iuliani:** Data curation (supporting); formal analysis (supporting); methodology (supporting); visualization (supporting); writing—review and editing (equal). **Sven Bergmann:** Conceptualization (equal); funding acquisition (lead); methodology (equal); project administration (lead); resources (lead); supervision (lead); writing—review and editing (equal).

## DECLARATION OF INTERESTS

The authors declare no competing interests.

## Supporting information


**Figure S1.** Schematic representation of the bidirectional Mendelian randomization (MR) analysis framework used in this study.
**Figure S2.** Distribution of median T2* and QSM for each sex.
**Figure S3.** Median T2* and QSM values by age.
**Figure S4.** Distribution of median T2* and QSM for each smoking status and sex.
**Figure S5.** Beta coefficients of smoking‐by‐sex interaction terms in linear regression models linking striatal iron and smoking.
**Figure S6.** Beta coefficients of smoking‐by‐age interaction terms in linear regression models linking striatal iron and smoking.
**Figure S7.** Right putamen QSM in former smokers by years since smoking cessation and packyears quartiles.
**Figure S8.** Gene‐level correlation between smoking and striatal iron, excluding genes previously associated with possible confounders (weekly alcohol consumption and serum iron).
**Figure S9.** Causality pathway from smoking to striatal iron, excluding genes previously associated with possible confounders (weekly alcohol consumption and serum iron).
**Figure S10.** Causality pathway from striatal iron to smoking, excluding genes previously associated with possible confounders (weekly alcohol consumption and serum iron).
**Figure S11.** Gene‐level correlation between smoking and striatal iron, using summary statistics from non‐overlapping samples (smoking GWAS without UK Biobank participants).
**Figure S12.** Causality pathway from smoking to striatal iron, using summary statistics from nonoverlapping samples (smoking GWAS without UK Biobank participants).
**Figure S13.** Causality pathway from striatal iron to smoking, using summary statistics from nonoverlapping samples (smoking GWAS without UK Biobank participants).

**Table S1.** Number and percentage of imputed values for each covariate.
**Table S2.** Number and percentage of outlier values excluded from the analysis.
**Table S3.** Robustness metrics of sensitivity analyses.
**Table S4.** Genes previously associated with potential confounders (drinks per week and serum iron).
**Table S5.** Descriptive statistics of non‐imaging variables in female, male, and all participants.
**Table S6.** Standardised effect sizes (β), standard errors (SE), and ‐log10(P‐values) of linear regression models linking striatal iron and smoking (n≈42k).
**Table S7.** Asymmetry between left and right accucumbers QSM association with smoking.
**Table S8.** Standardised effect sizes (β), standard errors (SE), and ‐log10(p‐values) of linear regression models linking striatal iron and smoking among former smokers (n≈42k).
**Table S9.** Phenotypic and genetic correlation between striatal iron and smoking initiation (‘ever smoked’).
**Table S10.** Gene‐level correlation between smoking and striatal iron performed using the PascalX cross‐GWAS coherence test.
**Table S11.** Mendelian randomisation estimates between smoking initiation and striatal iron.
**Table S12.** Mendelian randomisation sensitivity analyses testing model assumptions.
**Table S13.** Mendelian randomisation leave‐one‐out analysis using the inverse‐variance weighted method.
**Table S14.** Mendelian randomisation inverse‐variance weighted analysis with methodological variations.
**Table S15.** Gene‐level causality pathway from smoking to striatal iron performed using the PascalX ratio test.
**Table S16**. Gene‐level causality pathway from striatal iron to smoking performed using the PascalX ratio test.

## Data Availability

UK Biobank data are available upon successful application (https://www.ukbiobank.ac.uk/enable-your-research/apply-for-access). GWAS summary statistics used in this study are publicly available at https://www.fmrib.ox.ac.uk/ukbiobank/gwas_resources/ and https://open.oxcin.ox.ac.uk/ukbiobank/big40/ for brain traits, https://conservancy.umn.edu/items/ca7ed549-636b-41c0-ae79-97c57e266417 for smoking initiation and alcohol consumption, and https://www.decode.com/summarydata/ for serum iron. The code used to generate the presented results will be made available on GitHub upon publication (https://github.com/ot710/smoking_striatum_iron).
