# Supplementary material for: Bidirectional genetic and phenotypic links between smoking and striatal iron content involving dopaminergic and inflammatory pathways
Source: Addiction. 2026 Jan 31;121(6):1580–93. doi: 10.1111/add.70311 (PMC13155275; doi:10.1111/add.70311)
Supplement: Supplementary file 1 — Figure S1. Schematic representation of the bidirectional Mendelian randomization (MR) analysis framework used in this study. Figure S2. Distribution of median T2* and QSM for each sex. Figure S3. Median T2* and QSM values by age. Figure S4. Distribution of median T2* and QSM for each smoking status and sex. Figure S5. Beta coefficients of smoking‐by‐sex interaction terms in linear regression models linking striatal iron and smoking. Figure S6. Beta coefficients of smoking‐by‐age interaction terms in linear regression models linking striatal iron and smoking. Figure S7. Right putamen QSM in former smokers by years since smoking cessation and packyears quartiles. Figure S8. Gene‐level correlation between smoking and striatal iron, excluding genes previously associated with possible confounders (weekly alcohol consumption and serum iron). Figure S9. Causality pathway from smoking to striatal iron, excluding genes previously associated with possible confounders (weekly alcohol consumption and serum iron). Figure S10. Causality pathway from striatal iron to smoking, excluding genes previously associated with possible confounders (weekly alcohol consumption and serum iron). Figure S11. Gene‐level correlation between smoking and striatal iron, using summary statistics from non‐overlapping samples (smoking GWAS without UK Biobank participants). Figure S12. Causality pathway from smoking to striatal iron, using summary statistics from nonoverlapping samples (smoking GWAS without UK Biobank participants). Figure S13. Causality pathway from striatal iron to smoking, using summary statistics from nonoverlapping samples (smoking GWAS without UK Biobank participants). [file ADD-121-1580-s001.pdf]

Bidirectional genetic and phenotypic links between smoking and striatal iron content involving dopaminergic and inflammatory pathways - Supplementary Figures

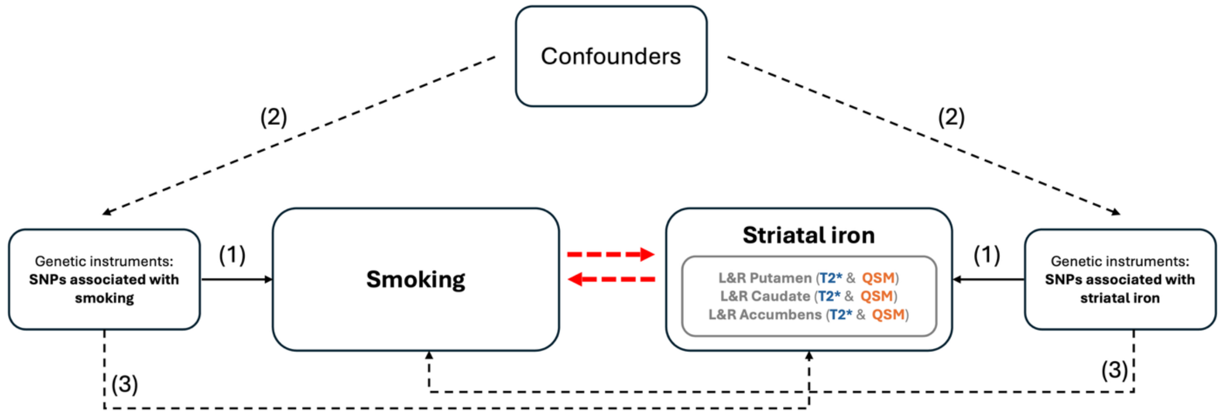

Figure S1. Schematic representation of the bidirectional Mendelian randomization (MR) analysis framework used in this study. Red arrows indicate the hypothesised causal relationships explored between the two traits, while black arrows represent the core MR assumptions: (1) genetic instruments are significantly associated with the exposure of interest, (2) genetic instruments are independent of any confounding factors, and (3) genetic instruments affect the outcome only via the exposure.

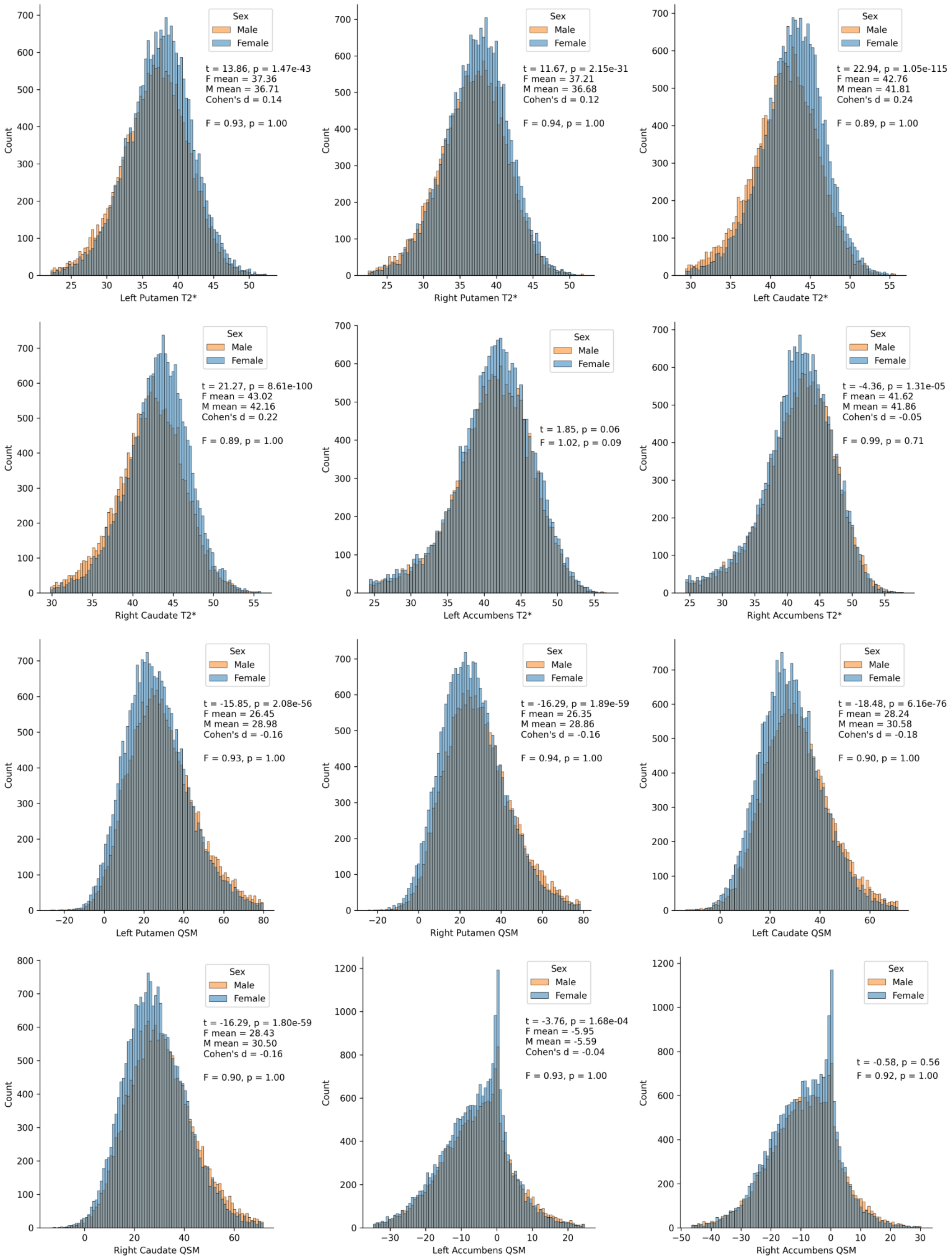

Figure S2. Distribution of median T2* and QSM for each sex. Male and female means were compared using two-sample *t*-tests. Respective means are indicated along with effect sizes (Cohen's *d*) when the *p*-value was <0.05. Male and female variances were compared using *F*-tests. F mean: female mean, M mean: male mean, QSM: quantitative susceptibility mapping.

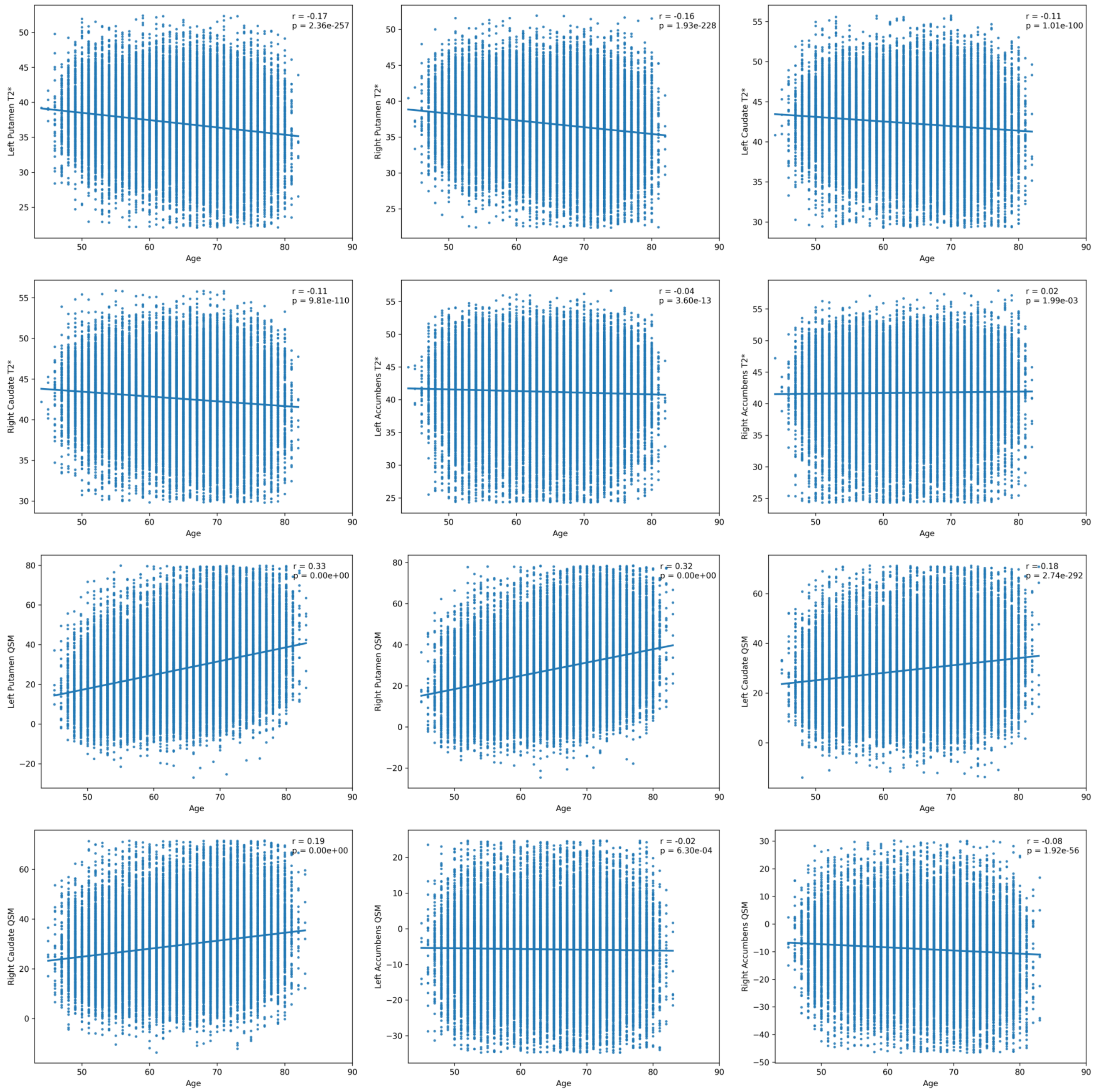

Figure S3. Median T2* and QSM values by age. Pearson's correlation coefficients and *p*-values are indicated. QSM: quantitative susceptibility mapping.

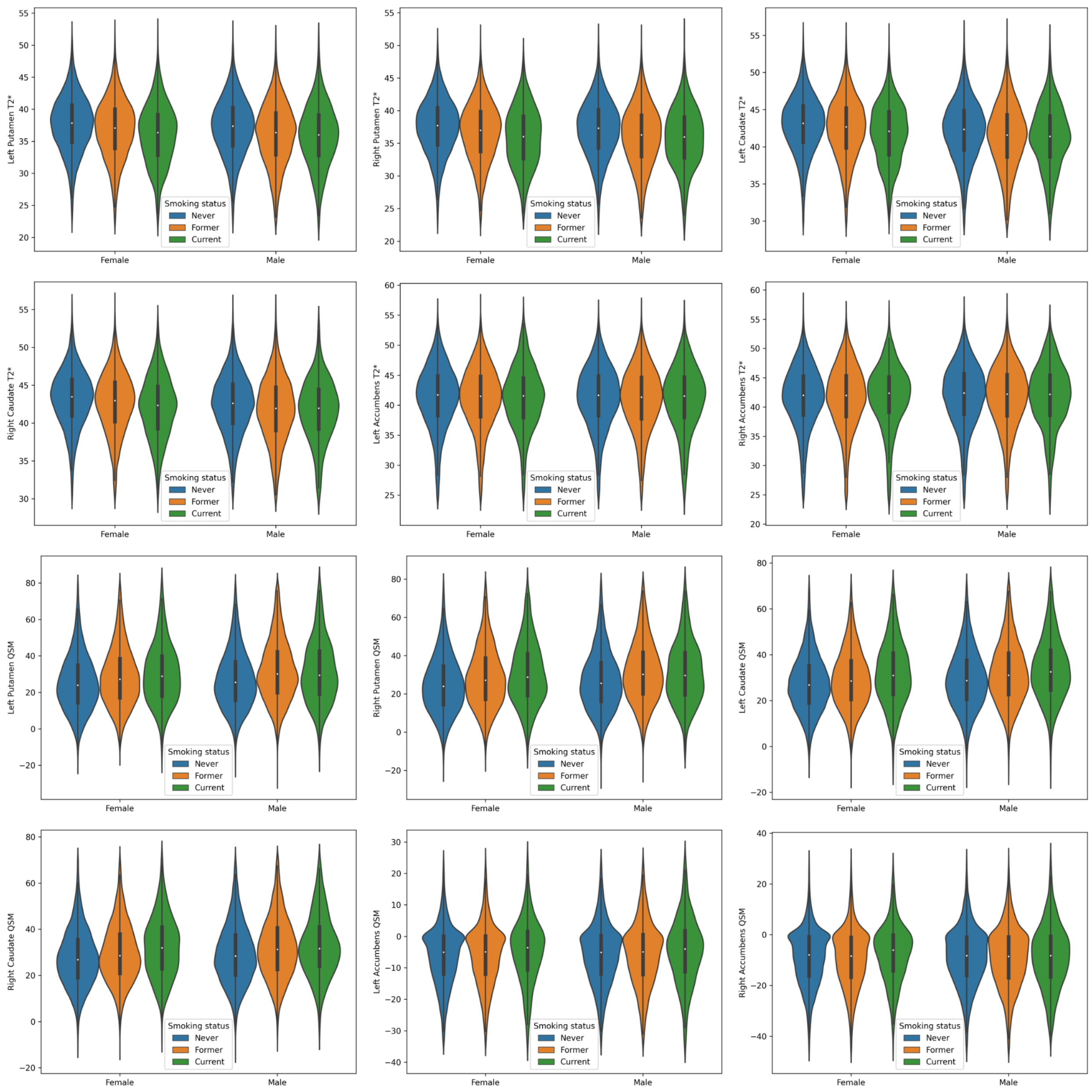

Figure S4. Distribution of median T2* and QSM for each smoking status and sex. No statistical tests were used to compare the groups. QSM: quantitative susceptibility mapping.

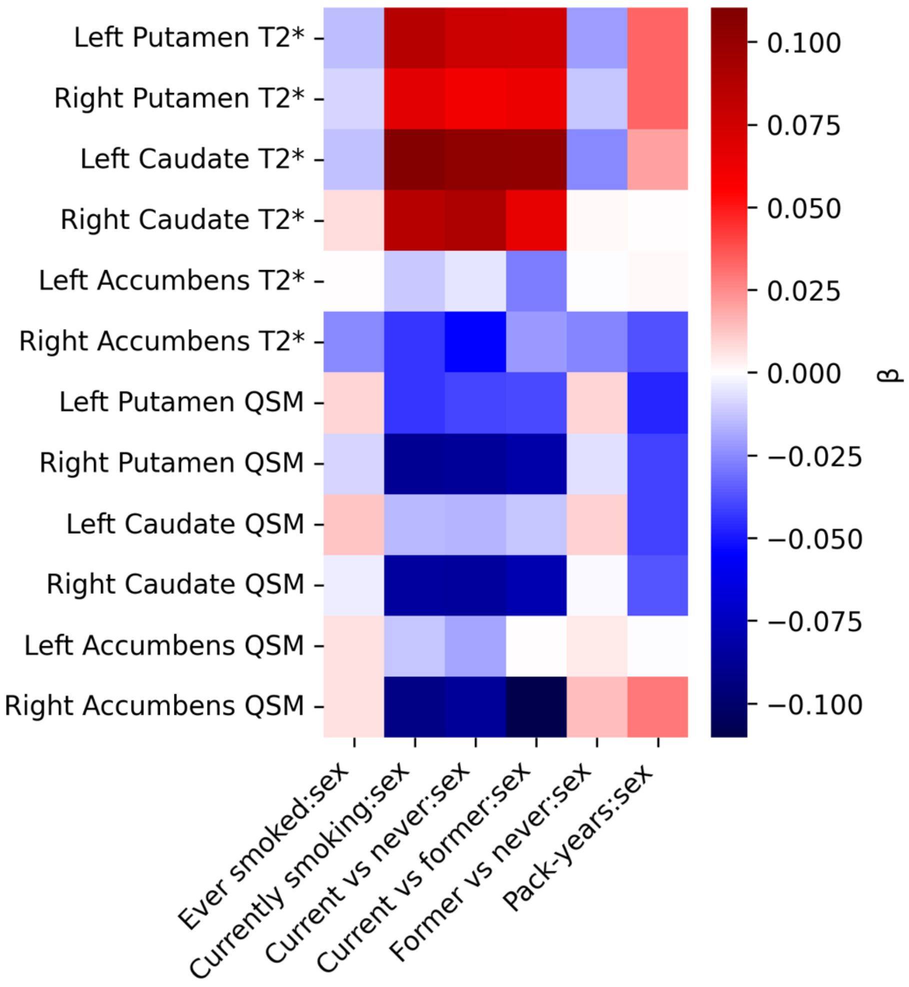

Figure S5. Beta coefficients of smoking-by-sex interaction terms in linear regression models linking striatal iron and smoking. Linear models also included main effects of smoking and sex, but only the interaction effects are shown here. None of them was statistically significant. QSM: quantitative susceptibility mapping.

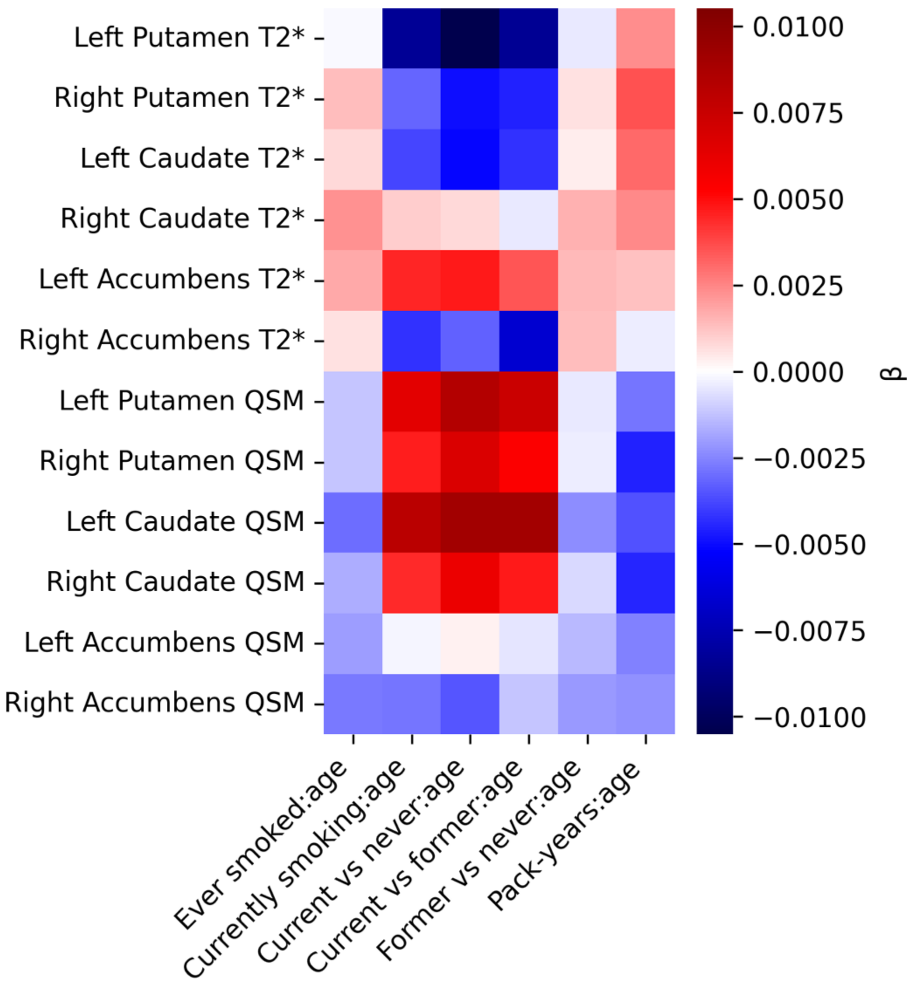

Figure S6. Beta coefficients of smoking-by-age interaction terms in linear regression models linking striatal iron and smoking. Linear models also included main effects of smoking and age, but only the interaction effects are shown here. None of them was statistically significant. QSM: quantitative susceptibility mapping.

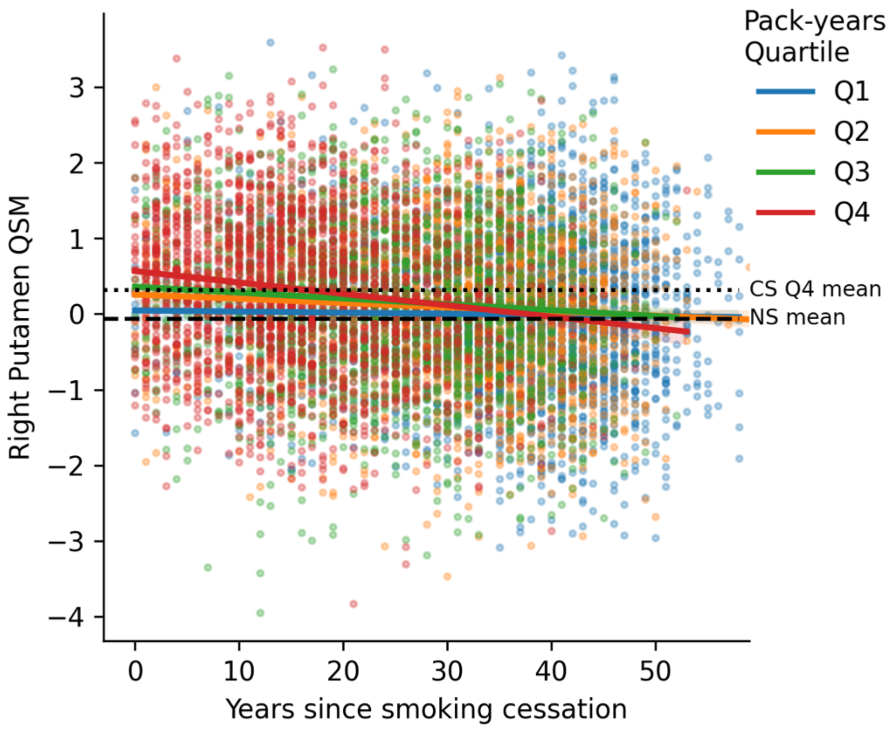

Figure S7. Right putamen QSM in former smokers by years since smoking cessation and pack-years quartiles. We show the right putamen QSM as an example, since a similar pattern was observed in all dorsal striatum regions (see Fig. 1b). The dashed line indicates the mean value of never smokers, while the dotted line, that of current smokers in the fourth pack-years quartile. Coloured lines represent regression lines of respective pack-years quartiles in former smokers. The interaction between years since smoking cessation and pack-years is made visible by the difference in slopes between quartiles: while the first quartile's QSM mean is very close to that of never smokers regardless of years since smoking cessation, the QSM mean of the fourth quartile is higher at 0 years since smoking cessation and similar to never smokers at about 40 years since smoking cessation. CS: current smokers, NS: never smokers, QSM: quantitative susceptibility mapping.

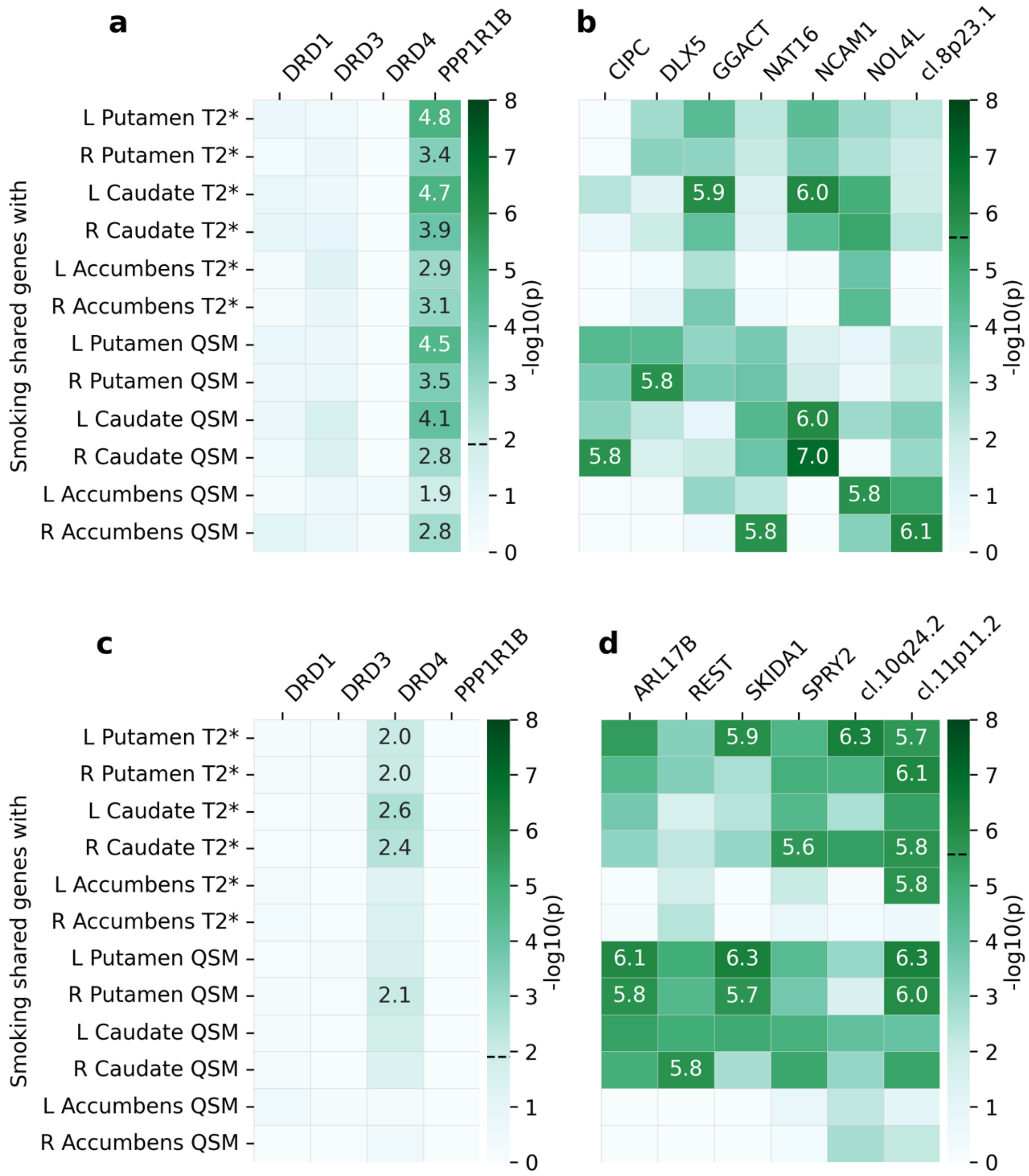

Figure S8. Gene-level correlation between smoking and striatal iron, excluding genes previously associated with possible confounders (weekly alcohol consumption and serum iron). We tested **(a, b)** positive and **(c, d)** negative correlations for **(a, c)** dopamine-related candidate genes and **(b, d)** an exhaustive set of 18 344 genes. –Log10(p) values are annotated for Bonferroni-significant pairs. Significance thresholds are indicated by dashed lines on the colour bars. L: left, R: right, QSM: quantitative susceptibility mapping. 'cl.' indicates gene clusters with their cytogenetic location; cl. 8p23.1: *C8orf74*, *PINX1*, *RP1L1*, *SOX7*; cl. 10q24.2: *AS3MT*, *CNNM2*; cl. 11p11.2: *GYLTL1B*, *PEX16*.

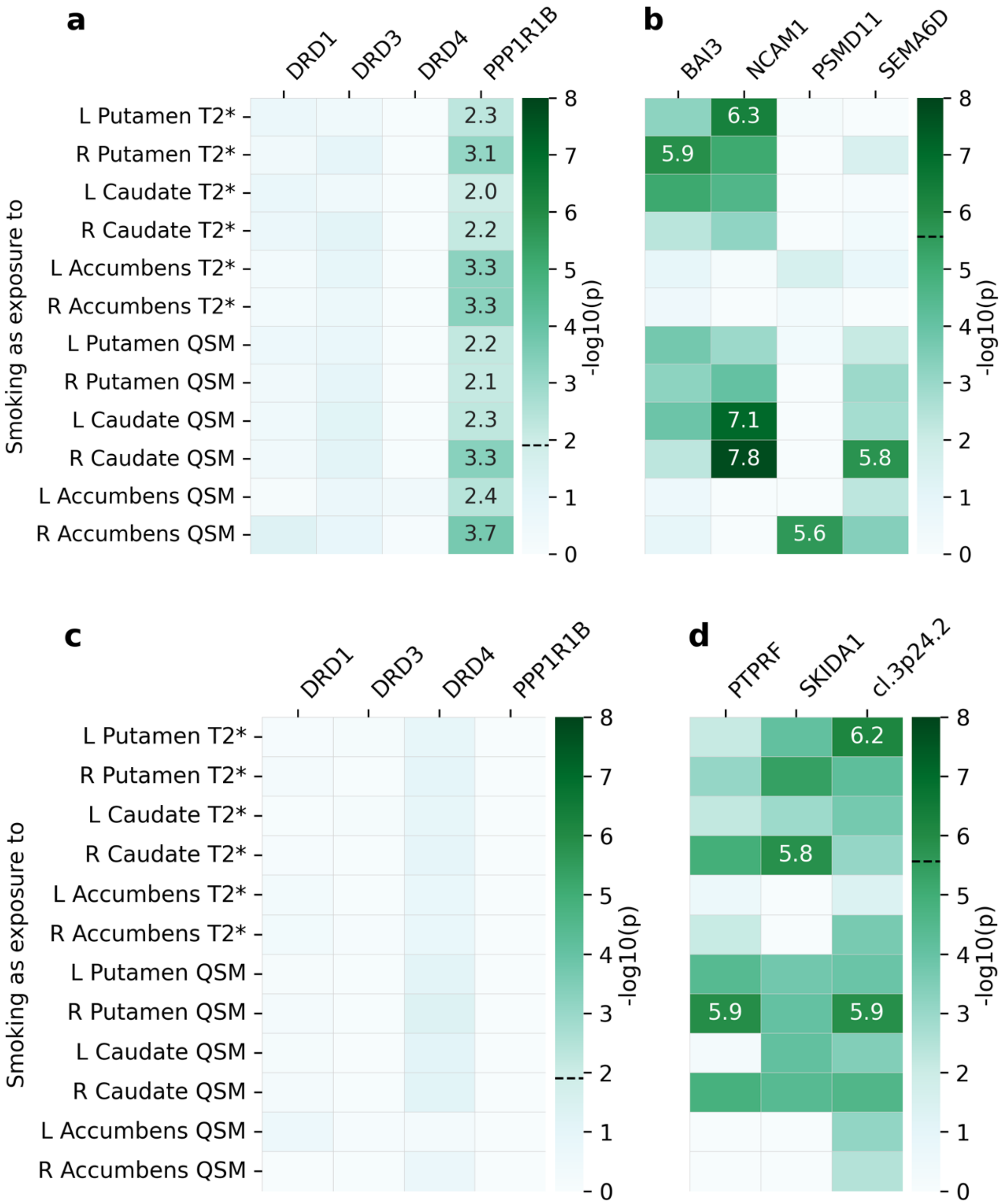

Figure S9. Causality pathway from smoking to striatal iron, excluding genes previously associated with possible confounders (weekly alcohol consumption and serum iron). We used the PascalX cross-GWAS ratio test for **(a, b)** positive and **(c, d)** negative causal associations in **(a, c)** dopamine-related candidate genes and **(b, d)** an exhaustive set of 18 344 genes. –Log10(p) values are annotated for Bonferroni-significant pairs. Significance thresholds are indicated by dashed lines on the colour bars. L: left, R: right, QSM: quantitative susceptibility mapping. 'cl.' indicates gene clusters with their cytogenetic location; cl. 3p24.2: *RARB*, *TOP2B*.

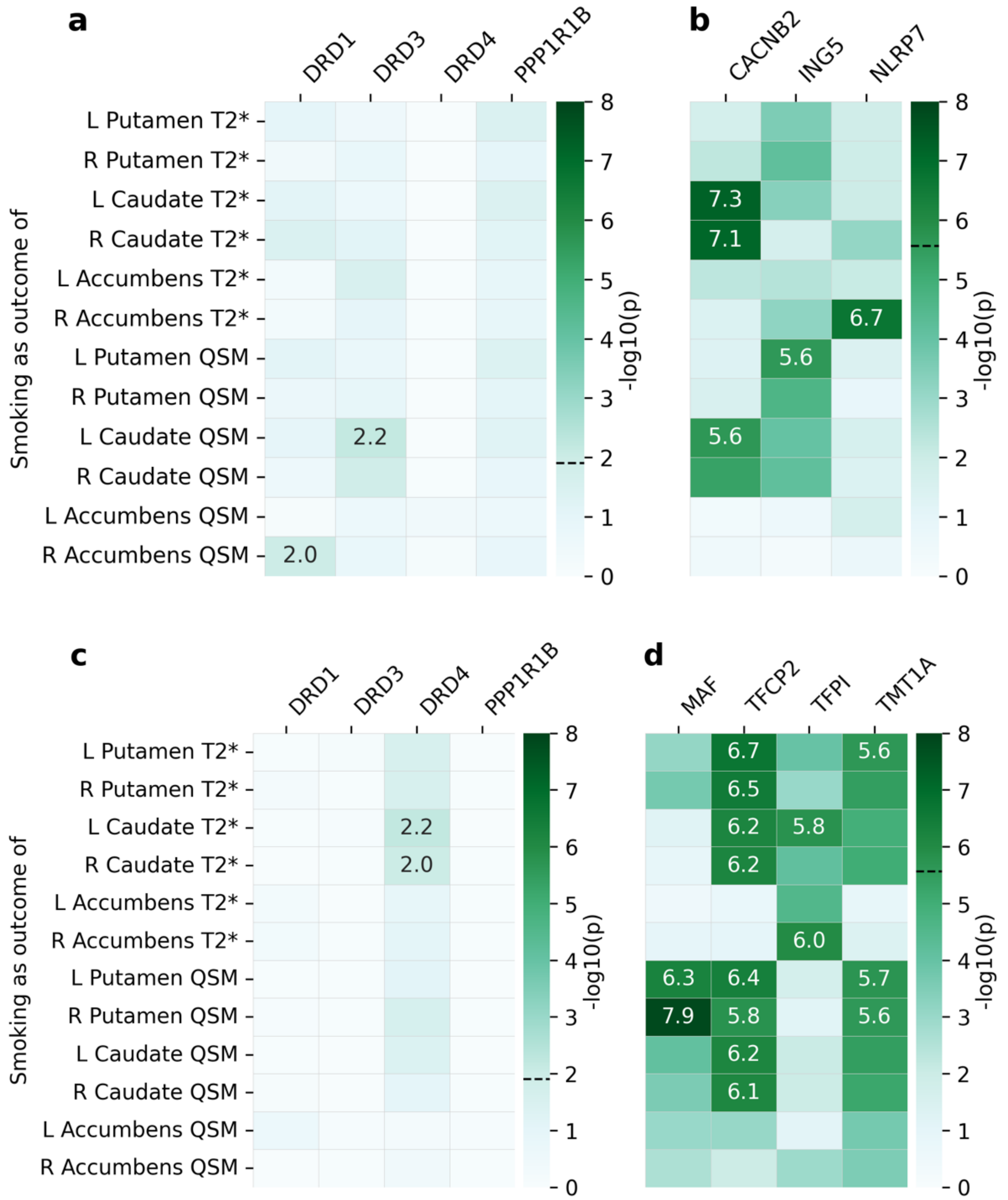

Figure S10. Causality pathway from striatal iron to smoking, excluding genes previously associated with possible confounders (weekly alcohol consumption and serum iron). We used the PascalX cross-GWAS ratio test for **(a, b)** positive and **(c, d)** negative causal associations in **(a, c)** dopamine-related candidate genes and **(b, d)** an exhaustive set of 18 344 genes. –Log10(p) values are annotated for Bonferroni-significant pairs. Significance thresholds are indicated by dashed lines on the colour bars. L: left, R: right, QSM: quantitative susceptibility mapping. 'cl.' indicates gene clusters with their cytogenetic location; cl. 12q13.12: *TFCP2, TMT1A*.

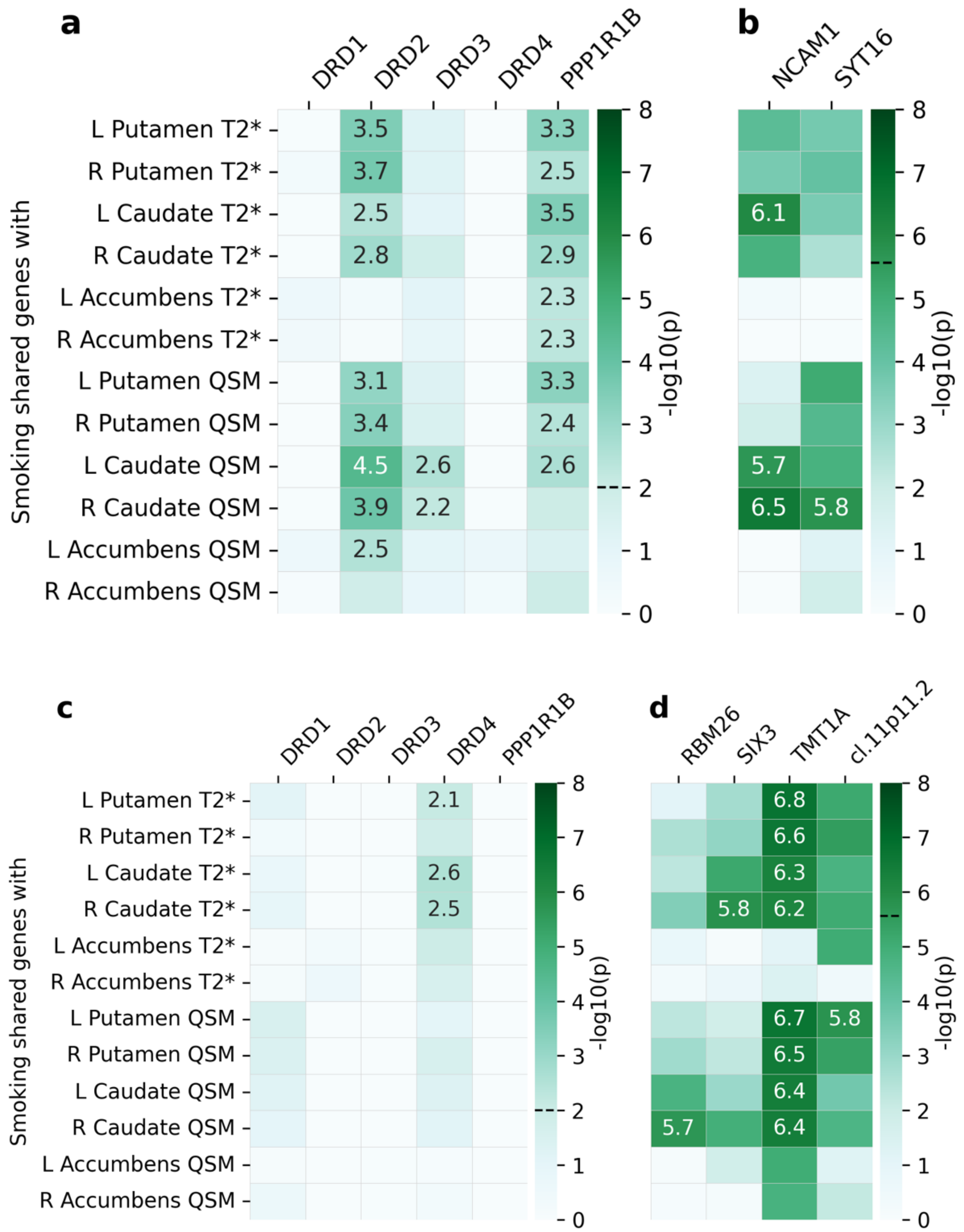

Figure S11. Gene-level correlation between smoking and striatal iron, using summary statistics from non-overlapping samples (smoking GWAS without UK Biobank participants). We tested **(a, b)** positive and **(c, d)** negative correlations for **(a, c)** dopamine-related candidate genes and **(b, d)** an exhaustive set of 18 344 genes. –Log10(*p*) values are annotated for Bonferroni-significant pairs. Significance thresholds are indicated by dashed lines on the colour bars. L: left, R: right, QSM: quantitative susceptibility mapping. 'cl.' indicates gene clusters with their cytogenetic location; cl. 11p11.2: *C11orf94*, *PEX16*.

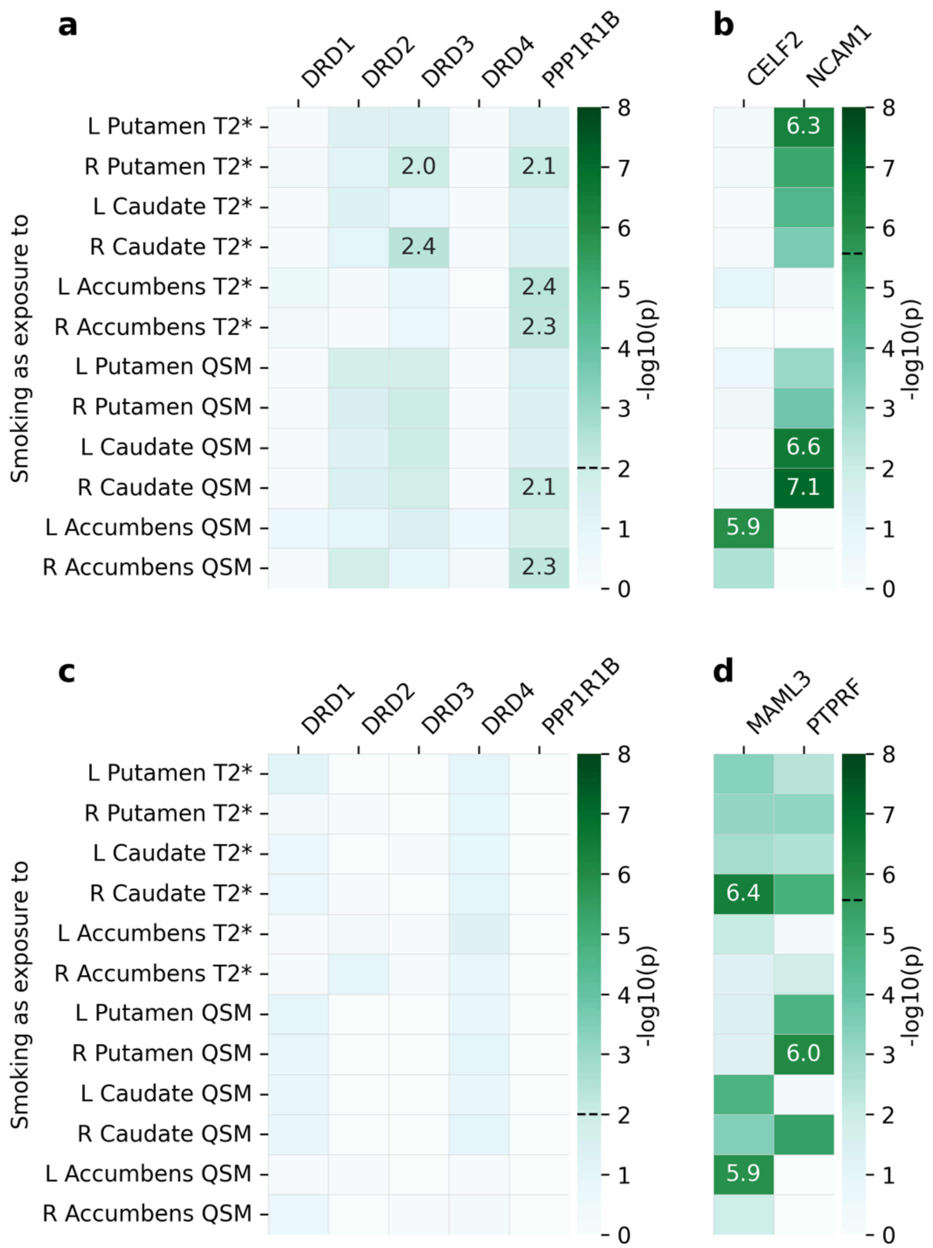

Figure S12. Causality pathway from smoking to striatal iron, using summary statistics from non-overlapping samples (smoking GWAS without UK Biobank participants). We used the PascalX cross-GWAS ratio test for **(a, b)** positive and **(c, d)** negative causal associations in **(a, c)** dopamine-related candidate genes and **(b, d)** an exhaustive set of 18 344 genes. –Log10(*p*) values are annotated for Bonferroni-significant pairs. Significance thresholds are indicated by dashed lines on the colour bars. L: left, R: right, QSM: quantitative susceptibility mapping.

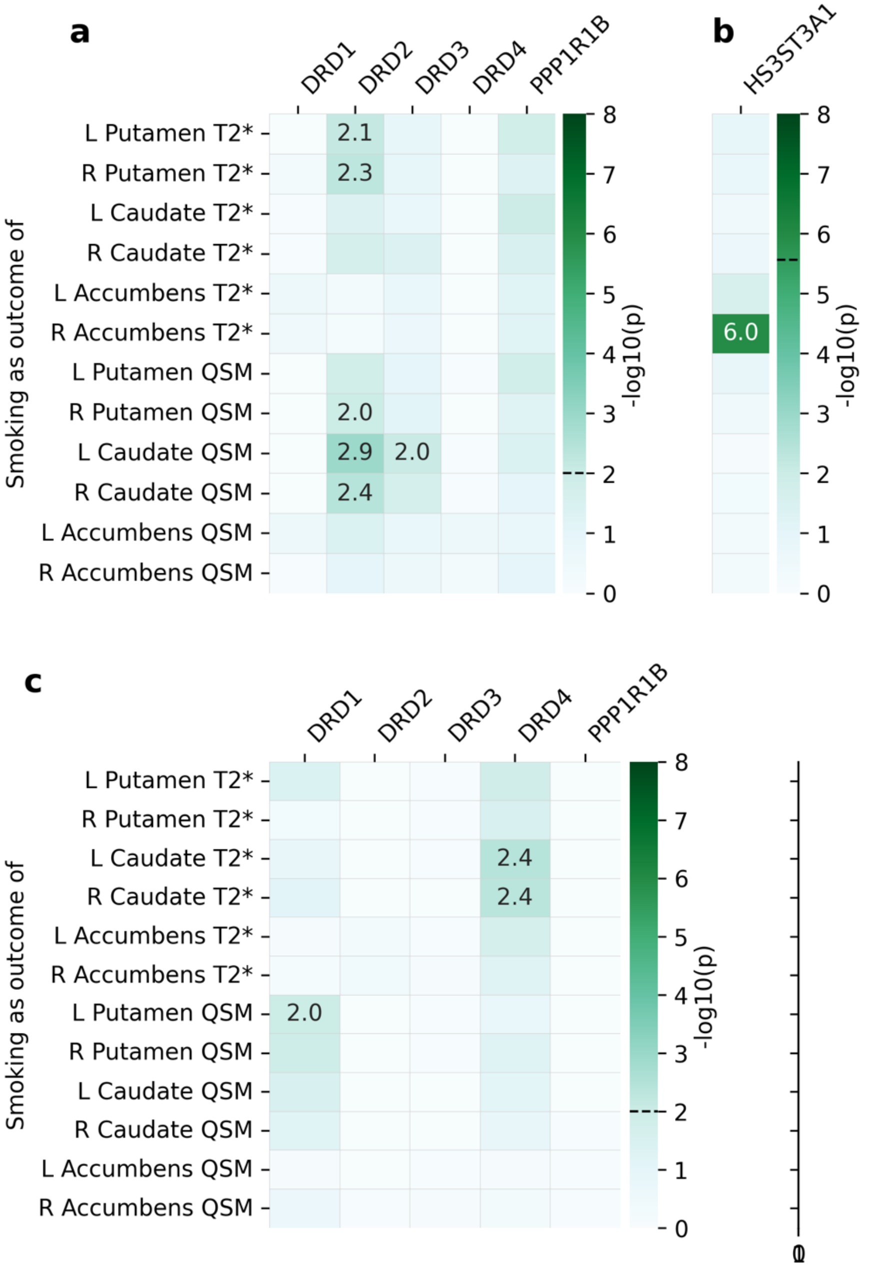

Figure S13. Causality pathway from striatal iron to smoking, using summary statistics from non-overlapping samples (smoking GWAS without UK Biobank participants). We used the PascalX cross-GWAS ratio test for **(a, b)** positive and **(c, d)** negative causal associations in **(a, c)** dopamine-related candidate genes and **(b, d)** an exhaustive set of 18 344 genes. –Log10(*p*) values are annotated for Bonferroni-significant pairs. Significance thresholds are indicated by dashed lines on the colour bars. L: left, R: right, QSM: quantitative susceptibility mapping.
